# Overview of the Design and Application of Dual-Signal Immunoassays

**DOI:** 10.3390/molecules29194551

**Published:** 2024-09-25

**Authors:** Xiaohua Ma, Yijing Ge, Ning Xia

**Affiliations:** 1 Department of Physical and Healthy Education, Nanchang Vocational University, Nanchang 330000, China; 2 Henan Key Laboratory of Biomolecular Recognition and Sensing, Shangqiu Normal University, Shangqiu 476000, China; 3 College of Chemistry and Chemical Engineering, Anyang Normal University, Anyang 455000, China

**Keywords:** immunoassays, dual signal, colorimetry, fluorescence, electrochemistry, surface-enhanced Raman spectroscopy

## Abstract

Immunoassays have been widely used for the determination of various analytes in the fields of disease diagnosis, food safety, and environmental monitoring. Dual-signal immunoassays are now advanced and integrated detection technologies with excellent self-correction and self-validation capabilities. In this work, we summarize the recent advances in the development of optical and electrochemical dual-signal immunoassays, including colorimetric, fluorescence, surface-enhanced Raman spectroscopy (SERS), electrochemical, electrochemiluminescence, and photoelectrochemical methods. This review particularly emphasizes the working principle of diverse dual-signal immunoassays and the utilization of dual-functional molecules and nanomaterials. It also outlines the challenges and prospects of future research on dual-signal immunoassays.

## 1. Introduction

Immunoassays based on highly specific antigen–antibody interactions are powerful analytical tools for the quantitative and qualitative detection of trace targets [1,2]. They have the advantages of high sensitivity, low cost, simple operation, excellent universality, and strong on-site monitoring ability. Immunoassays have been widely exploited in fields such as disease diagnosis, environmental monitoring, and food safety [3,4]. As one of the most commonly used methods, the enzyme-linked immunosorbent assay (ELISA) has been widely used in clinical detection of cancer biomarkers [5]. In order to achieve sensitive detection of targets in practical applications, many detection techniques are combined with ELISA. According to the type of output signal, immunoassays can be divided into colorimetric, fluorescent, chemiluminescence, surface-enhanced Raman spectroscopy (SERS), electrochemical, electrochemiluminescence (ECL), and photoelectrochemical (PEC) immunosensors [6,7]. However, most of the above-mentioned immunoassays are usually conducted in single–signal detection mode, which is easily influenced by various factors such as unstable experimental environments, non-standard experimental procedures, and differences between different operators, leading to abnormal fluctuations in signal intensity. Therefore, improving the accuracy and reliability of immunoassays has attracted the attention of many researchers.

Recently, dual-mode immunoassays combined with different intelligent detection mechanisms have attracted considerable attention for the detection of a single target due to their capability of self-correction and self-validation [8], making them excellent alternatives to conventional single–signal immunosensors [9]. Dual-signal immunosensors rely on the change in two signals caused by immunoreactions. Compared with the single–signal method, the two signals of dual-signal immunoassays are independent and there is no interference with each other. Dual-signal immunoassays have shown some unique advantages, such as wide detection range and high detection sensitivity. More importantly, they can avoid the fluctuations in the analysis data and enhance the mutual verification, ultimately improving the accuracy and reliability of immunosensing. Generally, dual-signal immunoassays involve two different signal reporters, dual-functional nanomaterials, and/or two techniques like magnetic separation and centrifugation [10,11]. For example, horseradish peroxidase (HRP) can catalyze the conversion of 3,3′,5,5′-tetramethylbenzidine (TMB) into blue oxidized TMB (TMBox) that can serve as both a chromogenic substrate and a fluorescence quencher [12]. With the development of nanotechnology, noble metal nanoparticles, especially gold nanoparticles (AuNPs), can be used as plasmonic chromogenic substrates and excellent fluorescence quenchers. The complexes formed between antigens and antibodies can be easily separated by magnetic or centrifugal separation, and the compositional difference between the supernatant and precipitate can be analyzed by different detection techniques [13].

Given the importance of dual-signal biosensors, several review papers have been reported focusing on the optical or electrochemical dual-signal biosensing [14,15,16,17,18,19,20]. For example, Sun et al. summarized the latest developments in integrated dual-mode optical biosensors for food safety [21]. Saleh et al. reported the development of dual-mode colorimetric and fluorescence biosensors for the determination of foodborne bacteria [22]. Hsu et al. reviewed the progress of electrochemical and optical dual-signal biosensors for environmental pathogens detection [23]. However, there are few reviews that systematically summarize the optical and electrochemical dual-signal immunoassays. In this work, the advances in the design and application of dual-signal immunoassays are comprehensively discussed, which are divided into three sections: optical dual-signal immunoassays (colorimetric–fluorescence, colorimetric–SERS, and fluorescence–SERS), electrochemical dual-signal immunoassays (electrochemical–PEC and electrochemical–ECL), and optical–electrochemical dual-signal immunoassays (colorimetric–electrochemical, fluorescence–electrochemical, colorimetric–PEC, and fluorescence–PEC).

## 2. Optical Dual-Signal Immunoassays

Colorimetric immunoassays have attracted growing attention in point-of-care testing (POCT) due to their unique advantages of cost-effectiveness, simple operation, and naked-eye signal readout [24]. They mainly rely on the enzyme or nanozyme-catalyzed chromogenic reactions of colorless substrates, such as TMB and 2,2′-azino-bis(3-ethylbenzothiazoline-6-sulfonic acid) (ABTS). Natural enzymes widely used in colorimetric immunoassays include HRP, glucose oxidase (GOx), and alkaline phosphatase (ALP) [25]. Some metal ions, such as Cu^2+^, Fe^2+^, and Ce^3+^, can catalyze the transformation of H_2_O_2_ into hydroxyl radicals via the famous Fenton reaction to subsequently oxidize organic chromogenic compounds [26,27]. Many nanomaterials with enzyme-mimicking properties have been used as nanozymes, such as metal oxides [28,29,30], metal–organic frameworks [31], MXenes [32], and carbon-based nanostructures [33,34]. However, colorimetric immunoassays may have inherent drawbacks of low detection sensitivity and selectivity. To address these issues, colorimetric analysis has been combined with other optical methods to improve accuracy and sensitivity, such as fluorescence and SERS. Two independent signals can be used for mutual verification, expanding the detection range and broadening the application scenarios.

### 2.1. Colorimetric–Fluorescence Dual-Signal Immunoassays

Fluorescence immunoassays have attracted great interest in chemical and biochemical analysis due to the merits of simple operation, high sensitivity, and excellent selectivity. Various materials have been used as fluorescent probes in immunoassays, including fluorescent proteins, organic dyes, semiconductor quantum dots (QDs), upconversion nanoparticles (UCNPs), noble metal nanoclusters, and carbon dots (CDs) [35,36]. However, there are still some shortcomings in the interference of background fluorescence from biomolecules in complex matrices. By coupling their respective advantages, qualitative/semi-quantitative detection in colorimetric mode and quantitative detection by fluorescence mode are expected [37,38]. There are two detection mechanisms to realize colorimetric and fluorescence immunoassays. The strategy mainly relies on the enzyme- or nanozyme-catalyzed chromogenic reactions to provide a colorimetric signal and modulate the change in fluorescence signal [39]. In addition, plasmonic noble metal nanomaterials can be used as chromogenic substrates to construct colorimetric/fluorescence biosensors. Generally speaking, the chromogenic or fluorescence reactions occur in a homogeneous solution efficiently, and the reaction process can be easily monitored by simple instruments. In order to further simplify the detection procedure and reduce the cost, lateral flow immunochromatographic assays (LFIAs) have been popularly developed using dual-functional (colorful and fluorescent) nanoparticles as signal reporters [40].

#### 2.1.1. Homogeneous Methods

Normally, the colored products generated from the chromogenic reactions exhibit a typical and strong absorption band in visual wavelength. When the absorption of colored products overlaps with the emission (or the excitation) of fluorescent probes, the signal of fluorophore will be quenched by the inner filter effect (IFE), realizing the colorimetric–fluorescence dual-signal detection [41,42,43]. Furthermore, if the colored product can also emit light, the target can be directly quantified by two readout signals without the introduction of extra fluorescent probes (Table 1) [44].

**Figure 1:**
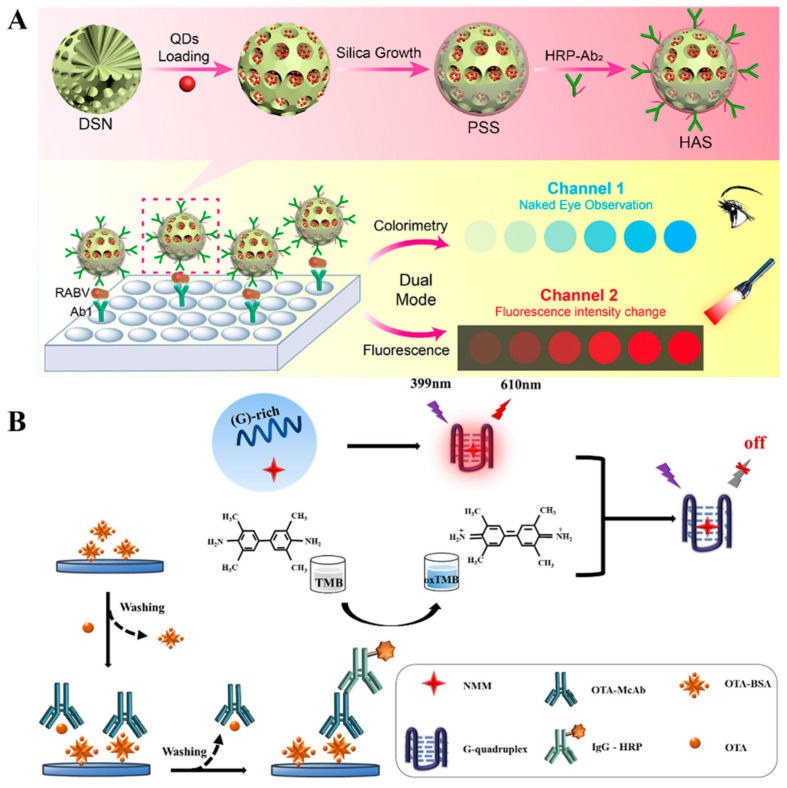
(**A**) Schematic illustration of a pomegranate-inspired silica nanotag-based immunoassay for colorimetric and fluorescence detection of rabies virus (RABV) [46]. Copyright 2020 American Chemical Society. (**B**) Schematic illustration of a colorimetric and fluorescence HRP-labeled immunoassay for OTA detection [48]. Copyright 2022 Elsevier.

Under the catalysis of enzymes or nanoenzymes, colorless TMB can be oxidized to blue TMBox, leading to changes in the signal of colorimetric detection [45]. Zhou et al. reported the colorimetric and fluorescence immunoassays of rabies virus (RABV) by using pomegranate-inspired silica (PSS) nanotags (Figure 1A) [46]. In this study, silica nanospheres (DSN) were used to load a large number of QDs, and then coated with a dense silica shell for the modification of HRP-conjugated antibodies. After the RABV nucleoprotein-based sandwich-like immunoreactions, HRP catalyzed the oxidation of TMB in the presence of H_2_O_2_, resulting in an obvious color change. Then, QDs in PSS produced a high fluorescence signal. Finally, this dual-signal immunoassay achieved the detection limits of 91 pg/mL (colorimetric) and 8 pg/mL (fluorescence). It has been reported that blue TMBox has fluorescence quenching ability through the IFE effect [47]. For this consideration, Xiao et al. reported colorimetric and fluorescence HRP-labeled immunoassays of Ochratoxin A (OTA) [48]. As shown in Figure 1B, the competitive immunoreaction was used to determine OTA in corn, oats, and rice samples. HRP catalyzed the oxidation of TMB into blue TMBox in the presence of H_2_O_2_. The absorption peak of TMBox matched with the emission peak of the G-quadruplex/N-methylmesoporphyrin IX (G4/NMM) complex, leading to the fluorescence quenching through the IFE effect. As a result, the detection limits of colorimetric and fluorescence immunoassays were found to be 4.316 pg/mL and 1.515 pg/mL, respectively.

**Figure 2:**
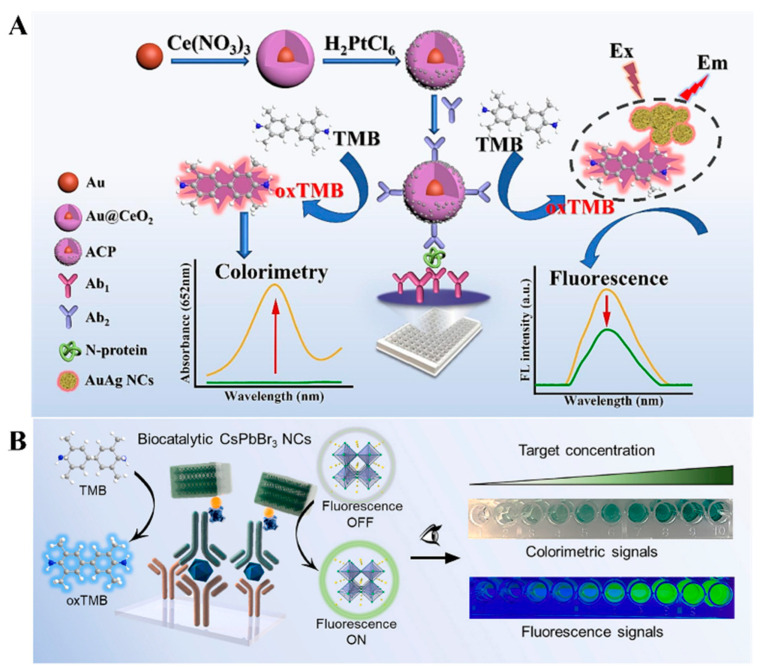
(**A**) Schematic illustration of a dual-mode immunoassay for SARS-CoV-2 N-protein detection based on Au@CeO_2_@Pt NPs [53]. Copyright 2023 Elsevier. (**B**) Schematic illustration of the dual-signal immunoassay for PSA detection using CsPbBr_3_ perovskite NCs as fluorescence and enzyme-like labels [54]. Copyright 2022 Elsevier.

Although natural enzymes have high catalytic activity and specificity, they exhibit the inherent drawbacks of high cost and low capacity in harsh environments. Since it was reported that Fe_3_O_4_ NPs exhibit peroxidase-like activity, a variety of nanomaterials has been used as excellent alternatives for enzymes to catalyze chromogenic and/or fluorogenic reactions in immunoassays [49]. Similar to natural enzymes, nanozyme-catalyzed chromogenic reactions can be combined with IFE to construct colorimetric and fluorescence immunosensors, such as CeO_2_@Au heterojunctions [50], Prussian blue-modified Fe_3_O_4_ nanoparticles [51], and MnO_2_ nanosheets [52]. Cao et al. developed a dual-signal immunoassay for SARS-CoV-2 N-protein detection based on Au@CeO_2_@Pt NPs (Figure 2A) [53]. In this study, Au@CeO_2_@Pt NPs were prepared via a layer-by-layer assembly strategy and many tiny PtNPs on the CeO_2_@Pt shell served as the catalytic sites. Au@CeO_2_@Pt NPs with HRP-like capability catalyzed the oxidation of colorless TMB into blue TMBox in the presence of H_2_O_2_, providing a visible colorimetric signal. The red-emitting fluorescence of AuAg NCs was efficiently quenched by TMBox via the IFE effect. The detection limits of the dual-signal immunoassays for SARS-CoV-2 N-protein detection were 0.062 ng/mL (colorimetric) and 0.036 ng/L (fluorescence), respectively. To date, several nanozymes have been demonstrated with both enzyme-like activity and fluorescence properties. For instance, Li et al. reported the colorimetric and fluorescence immunoassays of prostate-specific antigen (PSA) using CsPbBr_3_ perovskite nanocrystals (NCs) as fluorescence and enzyme-like labels (Figure 2B) [54]. Phospholipid-coated CsPbBr_3_ NCs used in this work exhibited excellent fluorescence and distinct peroxidase-like catalytic activity. Lipid shells around perovskite NCs improved their stability and antifouling ability against biological impurities. It produced a strong fluorescence signal and catalyzed the generation of blue TMBox to generate a colorimetric signal. The proposed dual-signal immunoassay for PSA detection achieved the detection limits of 0.29 ng/mL (colorimetric) and 0.081 ng/mL (fluorescence).

**Figure 3:**
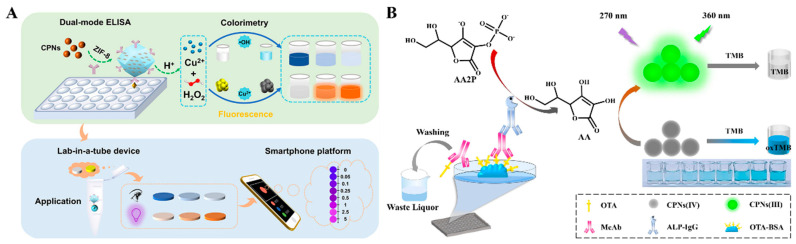
(**A**) Schematic illustration of the dual-mode immunoassay based on self-providing H_2_O_2_ and Cu^2+^ of CNPs@ZIF-8 for the detection of bisphenol A and development of the lab-in-a-tube device combined with a smartphone sensing platform [55]. Copyright 2022 American Chemical Society. (**B**) Schematic illustration of the fluorescence and colorimetric dual-mode immunoassay for detecting OTA based on cerium-based nanoparticles [58]. Copyright 2024 Elsevier.

Immunoassays with H_2_O_2_ as the oxidant have usually been limited to practical applications because of its low stability. Wei et al. reported a dual-mode immunoassay for bisphenol A detection using copper peroxide nanodot (CNPs)-loaded Zeolitic imidazolate framework-8 (ZIF-8, CNPs@ZIF-8) for self-supplying H_2_O_2_ (Figure 3A) [55]. CNPs@ZIF-8 was functionalized with antibodies against bisphenol A. After the immunoreaction was completed, CNPs located in the cavity of ZIF-8 were destroyed in an acid solution to release numerous H_2_O_2_ and Cu^2+^, which catalyzed the oxidation of TMB into blue TMBox by the Fenton reaction. Meanwhile, Cu^2+^ quenched the fluorescence of glutathione (GSH)-capped AuNCs (GSH-AuNCs). Furthermore, with the aid of antigen-modified immunomagnetic beads, a lab-in-a-tube device was developed with a smartphone platform. Unlike peroxidase, oxidase and its mimics can catalyze the oxidation of substrates in the absence of H_2_O_2_. For example, it was reported that ZIF-8-derived hollow Co/N-doped carbon nanotubes exhibit high oxidase-mimicking activity and can be employed as signal labels to catalyze the oxidation of TMB [56]. Ce^4+^ can catalyze the oxidization of TMB in the absence of H_2_O_2_, but its oxidase activity would be limited once Ce^4+^ was reduced to Ce^3+^. Based on this property, Chen et al. developed a Ce^4+^-modulated colorimetric and fluorescence immunoassays of OTA, in which Ce^4+^ was reduced by ALP-enzymatic product ascorbic acid (AA), and the resulting Ce^3+^ triggered the aggregation-induced emission (AIE) of AuNCs [57]. In addition, Zheng et al. developed a colorimetric and fluorescence immunoassay platform for OTA detection based on cerium-based nanoparticles (CNPs) (Figure 3B) [58]. In this work, CPNs(IV) with oxidase-like activity catalyzed the chromogenic reactions between TMB and H_2_O_2_. Following the ALP-labeled competitive immunoreactions, ALP catalyzed the conversion of ascorbic acid 2-phosphate (AAP) into AA, and CPNs(IV) were reduced into CPNs(III), leading to the inhibition of the chromogenic reactions. Meanwhile, CPNs(III) emitted a fluorescence signal.

**Figure 4:**
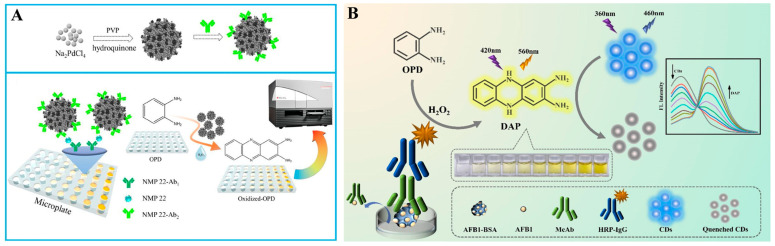
(**A**) Schematic illustration of the porous Pd nanoparticles-based fluorescence and colorimetric immunoassay for nuclear matrix protein 22 detection [62]. Copyright 2019 Elsevier. (**B**) Schematic illustration of the ratiometric fluorescence and colorimetric immunoassay for AFB1 detection based on OPD and CDs [66]. Copyright 2024 Elsevier.

*o*-Phenylenediamine (OPD) can be easily oxidized by certain metal ions or H_2_O_2_ under the catalysis of enzymes or nanozymes, and the product 2,3-diaminophenazine (OPDox) shows yellow color and intense orange-yellow fluorescence under the irradiation of ultraviolet light [59]. Therefore, it is feasible to develop colorimetric and fluorescence dual-signal immunoassays by using OPD as the substrate [60,61]. For example, Zhuge et al. developed a colorimetric and fluorescent immunoassay platform for the detection of nuclear matrix protein 22 based on nanozyme porous Pd NPs (Figure 4A) [62]. In this work, porous Pd NPs were prepared through a wet chemical reduction method using polyvinylpyrrolidone (PVP) as the surfactant and hydroquinone as a reductive agent. After the immunoreaction, Pd NPs modified with antibodies catalyzed the oxidation of OPD into OPDox in the presence of H_2_O_2_. The solution showed a strong fluorescence emission at 580 nm and the color changed from colorless to yellow. In addition, the GOx-triggered Fenton reaction and OPD oxidation have been combined to construct dual-signal immunoassays for the detection of danofloxacin in milk [63]. However, fluorescence immunoassays based on a single emission peak may suffer from the static or dynamic quenching that is caused by local fluorophore concentrations and environmental factors. To avoid this problem, other dyes or nanomaterials can be utilized as reference fluorophores in dual-mode immunoassays, realizing two emission signals under excitation at a single wavelength [64]. For instance, in the presence of a reference fluorophore, OPDox can quench its fluorescence via the IFE effect, enabling ratiometric sensing. Meanwhile, the color change from colorless to yellow can allow for the colorimetric detection of antigens. Based on this principle, Miao et al. developed a nanozyme-linked dual-mode immunoassay platform for the detection of cardiac troponin I, in which nanceria with HRP-like property catalyzed the conversion of OPD into yellow OPDox and quenched the fluorescence of graphitic carbon nitride quantum dots (g-C_3_N_4_ QDs) [65]. Xu et al. reported the colorimetric and ratiometric fluorescence immunoassays of Aflatoxin B1 (AFB1) detection based on OPD and CDs [66]. As shown in Figure 4B, OPD was oxidized by H_2_O_2_ to OPDox under the catalysis of HRP and the solution color became yellow. Meanwhile, the resulting OPDox quenched the fluorescence of CDs via the IFE effect. Based on the change in the fluorescence intensity of OPDox and CDs, a ratiometric fluorescence sensing platform was achieved.

As an isomer of OPD, *p*-phenylenediamine (PPD) can be oxidized into 2,5-diamino-N, N’-bis(p-aminophenyl)-l,4-benzoquinone di-imine (PPDox) by H_2_O_2_ under the catalysis of HRP, accompanied with the color change from colorless to amaranth [67]. Thus, PPD can be applied for the development of colorimetric and fluorescence dual-signal immunoassays. For example, in combination with conventional HRP-linked immunoassays, dual-signal detection platforms have been developed for the detection of *Alicyclobacillus acidoterrestris* and zearalenone, in which PPD was used as a chromogenic substrate and its oxidized form (PPDox) quenched the fluorescence by the IFE effect [68,69].

**Figure 5:**
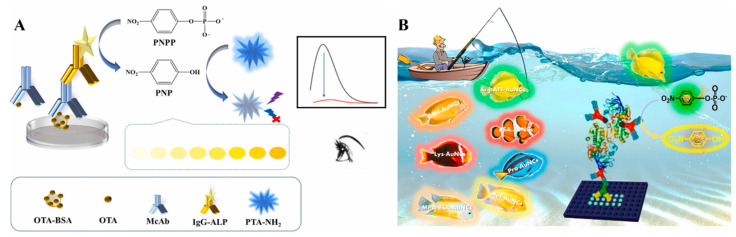
(**A**) Schematic illustration of the fluorescence and colorimetric immunoassay for OTA detection based on PPNP and 2-aminoterephthalic acid (PTA-NH_2_) [74]. Copyright 2024 Elsevier. (**B**) Schematic illustration of donors–acceptors selection to dual-signal immunoassay for AFB1 detection based on PPNP and AuNCs [76]. Copyright 2024 Elsevier.

ALP can effectively catalyze the dephosphorylation of phosphate esters into orthophosphoric acid and corresponding products [70]. Due to its high catalytic activity, excellent stability, and broad substrate specificity, ALP has been widely used as a reporter enzyme for immunoassays [71]. Its products can regulate the colorimetric or fluorescence signal of sensing systems with different principles, such as the generation of colored products, in situ generation of fluorescent molecules or nanomaterials by the enzymatic products, or the product-regulated aggregation of NPs [72]. ALP can catalyze the hydrolysis of *p*-nitrophenyl phosphate (PNPP) into yellow *p*-nitrophenol (PNP), which will further quench the fluorescence via the IFE effect [73]. Luo et al. reported a colorimetric and fluorescence immunoassay platform for the detection of OTA based on the fluorescence quenching of 2-aminoterephthalic acid (PTA-NH_2_) by ALP-enzymatic product PNP (Figure 5A) [74]. The dual-mode immunosensors were developed based on the color of PNP and the fluorescence of PTA-NH_2_. It is worth noting that the low spectral overlap extent between fluorophore and quencher will lead to weak IFE efficiency [75]. Aiming to achieve a large spectral overlap with PNP, Xiong et al. prepared different AuNCs with tunable multicolor fluorescence using amino acids, proteins, enzymes, and thiol molecules as stabilizing agents (Figure 5B) [76]. Under the systemically investigation, L-arginine and 6-aza-2-thiothymine-capped AuNCs were used as the fluorescence indicators for the ALP-linked immunoassays. ALP-catalytic product PNP effectively quenched the fluorescence of AuNCs, providing a colorimetric and fluorometric signal. The AuNCs/PNPP system was applied for of AFB1 detection with the detection limits of 0.16 ng/mL and 0.06 ng/mL, respectively.

**Figure 6:**
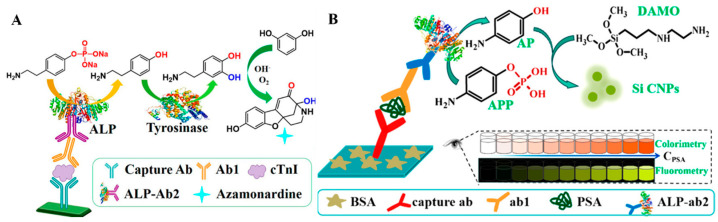
(**A**) Schematic illustration of a dual-mode immunoassay for the detection of cTnI based on the enzyme cascade-induced fluorogenic and chromogenic reaction [77]. Copyright 2018 American Chemical Society. (**B**) Schematic illustration of the dual-mode ELISA for PSA detection based on in situ generation of Si CNPs triggered by ALP [78]. Copyright 2020 American Chemical Society.

Based on the difference in the reactivity between substrates and their corresponding products, ALP-enzymatic products can trigger the in situ generation of fluorescent materials. Based on this fact, Zhao et al. reported a dual-mode immunoassay platform for the detection of cardiac troponin I (cTnI) based on the enzyme cascade-induced fluorogenic and chromogenic reactions using p-aminoethyl-phenyl phosphate disodium salt (PAPP) as the ALP-catalytic substrate [77]. As illustrated in Figure 6A, ALP catalyzed the conversion of PAPP into tyramine, which can be hydroxylated into dopamine under the catalysis of tyrosinase. The generated dopamine can react with resorcinol to form azamonardine in the Na_2_CO_3_ solution. The resulting solution showed a weak yellow color and strong blue fluorescence. By combining the enzyme catalysis with ALP-linked immunoassay, the dual-mode method for cTnI detection achieved detection limits of 60 fg/mL (colorimetric) and 15 fg/mL (fluorescence), respectively. Another major research hotpot involves the in situ formation of fluorescent nanomaterials based on the reduction ability of ALP-catalyzed products. For instance, Chen et al. developed a colorimetric and fluorescence immunoassay platform for PSA detection based on the ALP-induced in situ generation of silicon-containing NPs (Si CNPs) [78]. As displayed in Figure 6B, ALP catalyzed the transformation of 4-aminophenol phosphate (APP) into *p*-aminophenol (AP). Then, N-[3-(trimethoxysilyl)propyl]ethylenediamine (DAMO) was reacted with AP to produce colorimetric and fluorescence signals. PSA was detected with the detection limits of 9.6 ng/mL (colorimetric) and 4.1 ng/mL (fluorescence). With a similar protocol, Xie et al. reported dual-signal immunoassays of amantadine based on the AP-reduced in situ generation of carbon nanoparticles (CNPs) with yellow color and green fluorescence [79]. In this work, the linear ranges for colorimetric and fluorescence modes were 0.03–200 ng/g and 0.03–200 ng/g, respectively.

**Figure 7:**
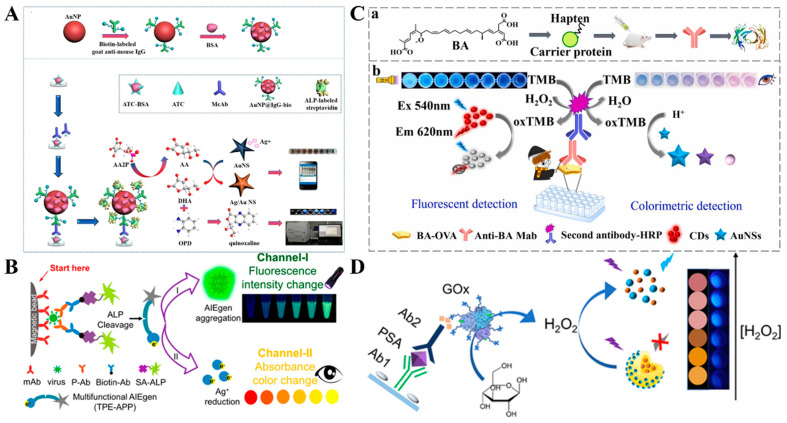
(**A**) Schematic illustration of a colorimetric–fluorescence dual-mode immunosensor for detection of chloroacetamide herbicides based on AuNSs and OPD [86]. Copyright 2021 American Chemical Society. (**B**) Schematic illustration of a dual-mode virion immunoassay based on AIE and in situ formation of silver shell on AuNPs [87]. Copyright 2018 American Chemical Society. (**C**) Schematic illustration of a TMBox-mediated colorimetric and fluorescence immunoassay for the determination of bongkrekic acid [90]. Copyright 2023 Elsevier. (**D**) Schematic illustration of a dual-mode colorimetric and fluorescence immunoassay for the detection of PSA with CD-encapsulated plasmonic core-satellite nanoprobes [92]. Copyright 2022 American Chemical Society.

Noble metal nanoparticles, mainly AuNPs and silver nanoparticles (AgNPs), possess molar extinction coefficients that are 1000-fold higher than the common organic dyes because of their unique localized surface plasmon resonance (LSPR) properties. The LSPR can be easily regulated by the size, shape, composition, dielectric environment, and interparticle distance of noble metal nanoparticles, resulting in shifts in maximum absorption peak and color change [80]. Therefore, noble metal nanoparticles have been widely used as chromogenic substrates or fluorescence quenchers for the development of colorimetric and fluorescence dual-signal immunoassays [81,82,83,84,85]. For example, Zha et al. presented a dual-mode immunosensor for the detection of chloroacetamide herbicides by using gold nanostars (AuNSs) and OPD as the signal reporters [86]. As shown in Figure 7A, AuNPs were modified with biotin-labeled IgG. After the competitive immunoreaction on the plate, ALP-labeled streptavidin was immobilized on the surface of AuNPs through the interaction between biotin and streptavidin. ALP catalyzed the conversion of AAP into AA, which triggered the reduction of Ag^+^ and caused the formation of a silver shell around AuNSs, leading to the visual color change. At the same time, AA was oxidized by Ag^+^ into DHA, which reacted with OPD to form fluorescent quinoxaline. In addition, Xiong et al. reported a dual-mode virion immunoassay platform based on the AIE effect and in-situ formation of silver shells on AuNPs (Figure 7B) [87]. In this work, a multifunctional water-soluble AIE luminogen (AIEgen) TPP-APP was used as the substrate of ALP. In the ALP-labeled immunoassay with the aid of immunomagnetic beads, ALP catalyzed the hydrolysis of TPP-APP into insoluble TPE-DMA, which aggregated in water to generate strong fluorescence. Meanwhile, TPE-DMA reduced Ag^+^ to produce a silver shell on AuNPs, providing a significant color change.

Besides the in-situ growth of nanoparticles, in situ etching of plasmonic nanoparticles is another typical example based on the shape-dependent property of LSPR [88,89]. TMBox can etch plasmonic nanomaterials to cause an obvious color change. Cao et al. reported a TMBox-mediated colorimetric and fluorescence immunoassay method for the determination of bongkrekic acid (Figure 7C) [90]. In this work, TMBox produced from HRP-based enzymatic catalysis quenched the fluorescence of CDs through the IFE effect. Meanwhile, TMBox oxidized gold nanostars (AuNSs) with the aid of CTAB, and the solution color changed from purple to blue for visual readout. Under the optimized conditions, the proposed dual-mode immunoassays achieved the detection limits of 8.4 ng/mL (colorimetric) and 5.7 ng/mL (fluorescence). By using polyethyleneimine-coated Prussian blue (PB) nanocubes as pH-switchable nanozymes, Liang et al. reported a AuNS-based colorimetric and fluorescence immunoassay platform for rosiglitazone detection [91]. In an acidic medium, PB nanocubes exhibited HRP-like activity and triggered the chromogenic reaction of Au nanostars with the TMB_ox_/CTAB system. In alkaline conditions, PB nanocubes with catalase-like activity catalyzed the decomposition of H_2_O_2_ into O_2_ to induce the aerobic oxidation of 4-chloro-1-naphthol (4-CN), leading to a decrease in the fluorescence signal. For instance, Gao et al. reported a dual-mode colorimetric and fluorescence immunoassay platform for the detection of PSA with CD-encapsulated plasmonic core-satellite nanoprobes (Figure 7D) [92]. In this study, the core-satellite nanoprobe, CDs/Ag-Au, was synthesized by a sequential in situ reduction strategy without additional reducing agents. The fluorescence of CDs was quenched by the Ag core, resulting in a very low background. GOx catalyzed the oxidation of glucose to produce H_2_O_2_ for etching the CDs/Ag-Au core-satellite nanoprobes. The dissolution of the Ag core resulted in an obvious LSPR change with a solution color change from yellow to pink. Meanwhile, CDs were released from the nanoprobes alongside a significant fluorescence recovery. The detection limits of the colorimetric and fluorescence modes were 2.3 and 0.84 pg/mL, respectively.

**Figure 8:**
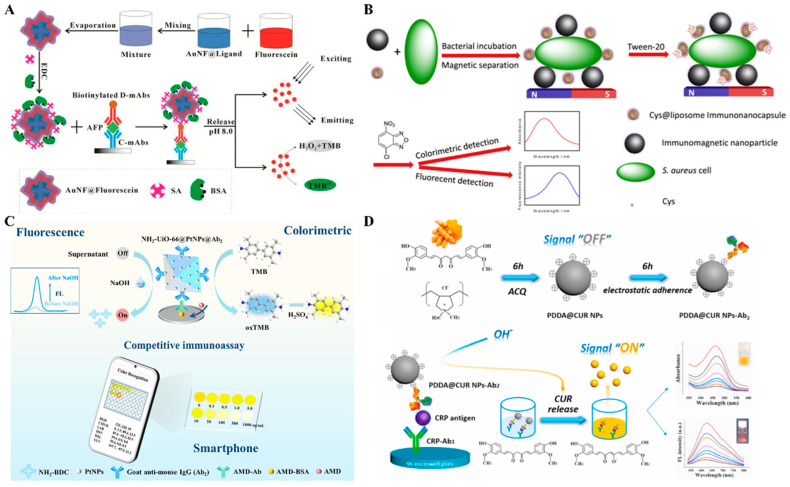
(**A**) Schematic illustration of a AuNF@Fluorescein-based colorimetric and fluorescence immunoassay for AFP detection [94]. Copyright 2018 Elsevier. (**B**) Schematic illustration of a colorimetric and fluorescence immunoassay for the detection of *S. aureus* based on Cys@liposome and immunomagnetic nanoparticles [97]. Copyright 2021 Elsevier. (**C**) Schematic illustration of a smartphone-assisted fluorescence–colorimetric immunosensing microarray for amantadine detection using NH_2_-UiO-66 stabilized PtNPs as dual-functional labels [99]. Copyright 2021 Elsevier. (**D**) Schematic illustration of the synthesis of CUR NPs and PDA@CUR and “lighting-up” CUR NPs-based dual-modal colorimetric and fluorescence immunoassay of CRP [101]. Copyright 2021 Elsevier.

Embedding sufficient signal reporters into one nanoparticle is a promising method for improving the detection sensitivity [93]. Zhou et al. reported a colorimetric–fluorescence dual-signal immunoassay strategy for AFP detection using fluorescein-loaded gold nanoflower (AuNF, AuNF@Fluorescein) as the signal label (Figure 8A) [94]. AuNF was modified with a thiolated carboxyl–ligand and further loaded with fluorescein via the hydrophobic interaction. The fluorescence of fluorescein was quenched by AuNF through the fluorescence resonance energy transfer (FRET) effect. In an alkaline solution (pH 8.0), fluorescein molecules were released from the hydrophobic wallet, leading to the recovery of the fluorescence signal. Meanwhile, fluorescein with intrinsic peroxidase-like activity catalyzed the oxidation of TMB by H_2_O_2_, generating a colorimetric signal. Liposomes, composed of a lipid bilayer and inner cavity, can embed a water-soluble or water-insoluble species into its inner cavity or its bilayer membrane. Thus, liposomes have been widely used as excellent nanocarriers in the fields of drug delivery and biosensing [95,96]. For instance, Deng et al. presented a colorimetric and fluorescence immunoassay platform for the detection of *Staphylococcus aureus* (*S. aureus*) based on L-cysteine (Cys)-encapsulated liposome (Cys@liposome) and immunomagnetic nanoparticles [97]. As shown in Figure 8B, *S. aureus* cells were captured by immunomagnetic nanoparticles and labeled with Cys@liposomes. After magnetic separation, Cys@liposomes were destroyed by Tween-20, leading to the release of a large amount of Cys molecules that reacted with 4-chloro-7-nitrobenzo-2-oxa-1,3-diazole to generate fluorescent and colorful adducts. 

Organic molecules with optical properties can be assembled into nanomaterials through various inter- or intra-molecule interactions and serve as signal labels in colorimetric and fluorescence immunoassays [98]. For example, 2-aminobenzene-1,4-dicarboxylic acid (NH_2_-BDC), a fluorescent organic ligand, can coordinate with metal ions and assemble into diverse MOFs. Li et al. reported a smartphone-assisted colorimetric–fluorescence immunosensing microarray for amantadine detection using NH_2_-UiO-66–stabilized PtNPs as dual-functional labels (Figure 8C) [99]. In this study, a Zr-based metal–organic framework (NH_2_-UiO-66) was prepared with NH_2_-BDC as the organic ligand and Zr^4+^ ion as the metal ion, and served as a nanocarrier for in situ growth of PtNPs. The fluorescence of NH_2_-BDC was quenched via the ligand–metal charge transfer effect between the metal ion and NH_2_-BDC. After the immunoreaction, PtNPs with high peroxidase-like activity catalyzed the chromogenic reaction between TMB and H_2_O_2_, providing an improved colorimetric signal. Meanwhile, the structure of NH_2_-UiO-66 could be destructed under alkaline hydrolysis, leading to the release of fluorescence ligand NH_2_-BDC due to the inhibition of ligand–metal charge transfer process. With the aid of the smartphone-assisted color recognition system, the dual-signal immunosensor showed a wide concentration range. Curcumin (CUR), as a kind of pH indicator, showed a reddish brown color because of the basic pH-induced allochroic effect. Benefitting from the features of drug delivery systems, Miao et al. reported a dual-mode immunoassay platform for colorimetric and fluorescence detection of cTnI based on the FRET and allochroic effects [100]. Under the stimulus of a basic solution, CUR molecules were released from MoS_2_ nanoflowers, restoring the fluorescence of CUR by blocking the FRET process. After that, this group reported a dual-mode immunoassay platform by using CUR NPs as the signal labels to avoid the use of other nanomaterials (Figure 8D) [101]. Due to the poor water solubility, CUR molecules quickly self-assembled into CUR NPs in the presence of cationic poly(diallyldimethylammonium chloride) (PDDA, PDDA@CUR NPs). The PDDA@CUR NPs modified with antibodies showed a negligible fluorescence signal in neutral water because of the aggregation-induced quenching effect. After treatment with basic water, numerous CUR molecules were rapidly released into the solution and transformed from keto form to hydrophilic enol anions. The keto–enol conversion of CUR led to the fluorescence recovery and the change in the solution color from colorless to orange.

**Table 1:** Performances of colorimetric–fluorescence dual-signal homogeneous immunoassays.

Signal Label	Target	Linear Range	Detection Limit	Ref.
HRP/QDs@PSS	RABV	0.12–23.4 and 0.012–46.8 ng/mL	91 and 8 pg/mL	[46]
HRP	OTA	0.049–1.563 and 0.04–25 ng/mL	4.316 and 1.515 pg/mL	[48]
Fe-MOFs	PSA	1–20 and 0–15 ng/mL	180 pg/mL	[49]
CeO_2_@Au	T-2 toxin	0.1–1 and 0.005–0.7 ng/mL	8.52 and 20.11 pg/mL	[50]
MPBNs	*S. aureus*	0.01–100,000 CFU/μL	0.1 CFU/μL	[51]
B-CDs@SiO_2_@MnO_2_	DEP	0.05–100 ng/mL	3.4 and 2.7 pg/mL	[52]
CsPbBr3 NCs	PSA	0.1–15 and 0.01–80 ng/mL	290 and 81 pg/mL	[54]
CPNs@ZIF-8	BPA	0–5 ng/mL	18 and 8 pg/mL	[55]
Co/NCNT	OTA	0.001–10 ng/mL	0.21 and 0.17 pg/mL	[56]
ALP	OTA	10–45 and 2.89–34.72 ng/mL	0.81 and 0.62 ng/mL	[57]
ALP	OTA	14–300 and 4.69–37.5 ng/mL	962 and 404 pg/mL	[58]
porous Pd NPs	NMP22	0.001–0.5 ng/mL	0.35 and 0.31 pg/mL	[62]
GOx	danofloxacin	1–10 and 1–25 ng/mL	648 and 337 pg/mL	[63]
nanoceria	cTnI	0.001–10 ng/mL	227 and 413 fg/mL	[65]
HRP	AFB1	0.75–1.8 and 0.75–1.8 ng/mL	62 and 13 pg/mL	[66]
HRP	*A. acidoterrestris*	4.8 × 10^2^–4.8 × 10^7^ CFU/mL	4.8 × 10^2^ CFU/mL	[68]
HRP	ZEN	0.048–3.125 and 0.012–3.125 ng/mL	11 pg/mL and 19 pg/mL	[69]
ALP	ZEN	7.5–20 and 7.5–17.5 ng/mL	7.22 and 36 pg/mL	[73]
ALP	OTA	0.39–25 and 1.56–50 ng/mL	70 and 570 ng/mL	[74]
ALP	AFB1	0.19–0.32 and 0.09–0.41 ng/mL	0.16 and 0.19 ng/mL	[76]
ALP	cTnI	0.2–80 and 0.05–4 ng/mL	60 and 15 ng/mL	[77]
ALP	PSA	0.02–28 and 0.02–20 ng/mL	4.1 and 9.6 pg/mL	[78]
ALP	amantadine	0.03–200 and 0.03–200 ng/g	47 and 60 ng/g	[79]
Au@CD	ferritin	1–160 and 1–120 ng/mL	20 and 64 ng/mL	[81]
ALP-AuNPs	ATC	0.63–84.59 ng/mL	1.2 and 0.44 ng/mL	[86]
ALP	EV71 virions	1340–134,000 and 1.67–2505 copies/μL	868.4 and 1.4 copies/μL	[87]
Cu_2_O@Fe(OH)_3_	OTA	0.001–10 ng/mL	0.83 and 0.56 pg/mL	[88]
HRP	BA	0–100 ng/mL	8.4 and 5.7 ng/mL	[90]
PBNCs@PEI	RSG	0.5–1000 pg/mL	95 and 63 fg/mL	[91]
GOx	PSA	0.005–20 ng/mL	2.3 and 0.84 ng/mL	[92]
QDs/ZnS NSs	AFP	0.05–12 ng/mL	7 and 10 pg/mL	[93]
AuNF@Fluorescein	AFP	5–5000 and 0.01–10 pg/mL	17.7 and 0.029 fg/mL	[94]
Cys@liposome	*S. aureus*	40–4000 and 4–4000 CFU/mL	10 and 1 CFU/mL	[97]
NH_2_-UiO-66@PtNPs	amantadine	0.1–1000 ng/mL	69 and 2.2 pg/mL	[99]
PDDA@CUR NPs	CUR	0.1–2 ng/mL	43 and 38 fg/mL	[101]

Abbreviation: HRP, horseradish peroxidase; QDs, quantum dots; PSS, pomegranate-shaped silica nanospheres; RABV, rabies virus; OTA, ochratoxin A; Fe-MOFs, Fe(III)-containing metal–organic frameworks; PSA, prostate-specific antigen; MPBNs, magnetic Prussian blue nanolabels; *S. aureus*, *Staphylococcus aureus*; B-CDs@SiO_2_@MnO_2_, blue carbon dots@ SiO_2_@MnO_2_; DEP, diethyl phthalate; CsPbBr3 NCs, CsPbBr_3_ perovskite nanocrystals; CPNs, copper peroxide nanodots; ZIF-8, zeolitic imidazolate framework-8; BPA, bisphenol A; Co/NCNT, Co nanoparticle/N-doped carbon nanotubes; ALP, alkaline phosphatase; NMP22, nuclear matrix protein 22; GOx, glucose oxidase; cTnI, cardiac troponin I; *A. acidoterrestri*, *Alicyclobacillus acidoterrestris*; AFB1, Aflatoxin B1; ZEN, zearalenone; Au@CD, gold@carbon dot; AuNPs, gold nanoparticles; ATC, acetochlor; EV71, enterovirus 71; Cu_2_O@Fe(OH)_3_, Cu_2_O@Fe(OH)_3_ yolk–shell nanocages; BA, bongkrekic acid; PBNCs@PEI, polyethyleneimine-coated Prussian blue nanocubes; RSG, an illegal additive; QDs/ZnS NSs, ZnS nanospheres modified with CdTe quantum dots; AFP, alpha-fetoprotein; Cys, L-cysteine; PtNPs, Platinum nanoparticles; NH_2_-UiO-66, Zr-based metal–organic framework; PDDA@CUR NPs, poly(diallyldimethylammonium chloride)-capped curcumin nanoparticles; CUR, curcumin.

#### 2.1.2. LFIA Methods

LFIAs are simple paper-based point-of-care (POC) testing devices that integrate the working principles of chromatography and immunochemical reactions [102,103,104]. Compared with the traditional ELISA, LFIAs exhibit the advantages of high simplicity, rapid analysis, portability, equipment-free, and cost-effectiveness. They have been popularly used in clinical diagnostics, food safety, and environmental monitoring [40,105,106,107]. The traditional colorimetric LFIAs mainly refer to the use of 10~30 nm AuNPs as nanolabels, and yet suffer from the inherent disadvantages of low color intensity and limited matric tolerance [108]. Fluorescent signals can be introduced into colorimetric LFIAs for more accurate quantitative analysis (Table 2) [109,110,111].

**Figure 9:**
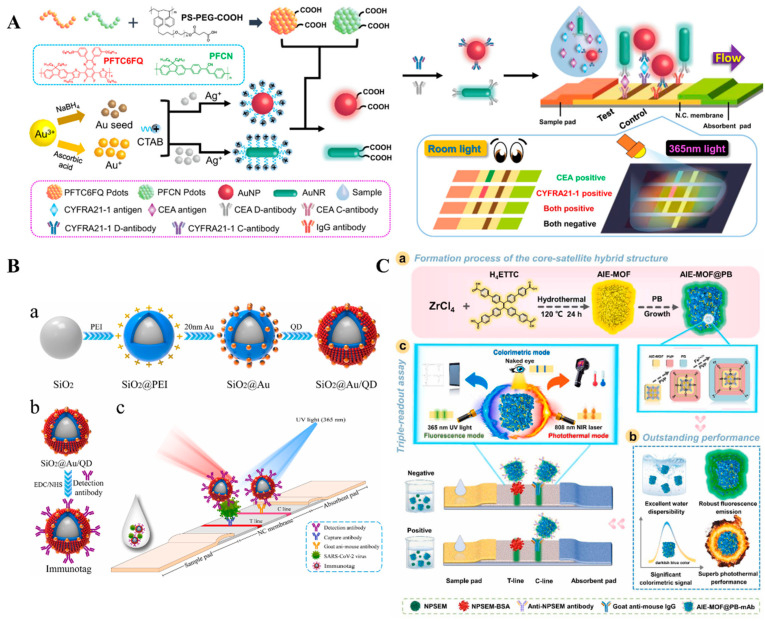
(**A**) Schematic illustration of the design of the Au@Pdot-based LFIA for the detection of CYFRA21-1 and CEA [114]. Copyright 2021 American Chemical Society. (**B**) Schematic illustration of (**a**) sequential process for fabricating dual-functional SiO_2_@Au/QD fluorescent labels; (**b**) preparation of S protein-conjugated SiO_2_@Au/QD labels; (**c**) schematic of a dual-functional LFIA biosensor [119]. Copyright 2022 Elsevier. (**C**) (**a**) Schematic illustration of AIE-MOF@PB hybrid structure; (**b**) the outstanding performances for AIE-MOF@PB; (**c**) triple-signal immunoassay of AIE-MOF@PB-LFIA [123]. Copyright 2022 Elsevier.

In LFIAs, AuNPs not only serve as signal labels for colorimetric assays but also as nanocarriers for photoluminescent materials [112]. For example, You et al. reported a colorimetric and fluorescent LFIA using polymer dot (Pdot)-modified AuNRs as multifunctional tags [113]. Then, they synthesized two types of Pdot-based nanocomposites for the dual-signal determination of carcinoembryonic antigen (CEA) and cytokeratin 19 fragment (CYFRA 21-1) [114]. As shown in Figure 9A, two types of Pdots with bright red and blue-green fluorescence were loaded by AuNPs and AuNRs, respectively. The formed Au@Pdot nanocomposites encompassed the merits of both plasmonic Au nanomaterials (i.e., larger extinction coefficient) and Pdots (i.e., strong fluorescence signal). As a result, the dual-mode LFIA method achieved detection limits of 0.07 for CYFRA21-1 and 0.12 ng/mL for CEA. Meanwhile, Pan et al. used the hybrids of fluorescent GSH-capped bimetallic nanoclusters and PEI-modified AuNPs as signal tags to realize the colorimetric and fluorescence detection of dicofol [115].

Nanomaterials with a large specific surface area can be used as the nanocarriers to load AuNPs and fluorescent species simultaneously for the preparation of dual-functional tags in LIFAs. Silica (SiO_2_) nanoparticles have been widely used as nanocarriers for signal amplification due to their excellent characteristics of adjustable size, ease of functionalization, and good stability [116,117,118]. Han et al. reported a colorimetric and fluorescence LFIA method for the determination of SARS-CoV-2 (Figure 9B) [119]. A mixed single-layer shell of 20 nm AuNPs and QDs was formed on the surface of a SiO_2_ core. AuNPs produce a strong colorimetric signal that can be directly observed without complicated instruments. QDs provided a strong fluorescence signal for a more sensitive quantification of the SARS-CoV-2 S antigen. Moreover, two-dimensional (2D) materials with larger specific surface areas, such as graphene oxide [120] and molybdenum disulfide nanosheet [121], have been employed to carry both AuNPs and QDs for dual-mode LFIAs. Magnetic nanoparticles with superparamagnetic properties can allow for the magnetic enrichment of a target in complex samples. Fang et al. used fluorescent microsphere/antibody-loaded magnetic nanobeads as trifunctional tags for colorimetric and fluorescence detection of sulfamethazine [122]. The linear ranges of the dual-signal LFIAs were 1–100 ng/mL (colorimetric) and 0.033–33 ng/mL (fluorescence), respectively. However, the co-assembly of AuNPs and QDs may result in fluorescence quenching due to the FRET process.

In recent years, nanomaterials with fluorescent and deep colorimetric characteristics have received widespread attention, especially those with AIE properties. For instance, Cheng et al. reported a triple-signal LFIA for the detection of nitrofurazone metabolites using PB-coated AIE-MOF (Figure 9C) [123]. In this work, the AIEgen of ethene-1,1,2,2-tetrayl)tetrakis(([1,1′-biphenyl]-4-carboxylic acid) was used as the organic ligand to synthesize AIE-MOF and the in situ coating with PB (AIE-MOF@PB). Owing to the combination of AIE-MOF and PB, the prepared tag exhibited a darkish blue color, yellow fluorescence, and excellent photothermal conversion efficiency, thus generating colorimetric, fluorescence, and photothermal signals in the LIFA. As a result, the detection sensitivity of this method was at least 5-fold higher than that of conventional AuNP-based LFIA. At the same time, Fan et al. reported a colorimetric and fluorescence LFIA for the detection of C-reactive protein using one-component dual-signal AIE nanobeads [124]. In this study, the red AIEgens with both colorimetric and fluorescent responses were embedded in polymer nanobeads and used as dual-functional tags. The AIE nanobead-based LFIA achieved the detection limits of 8.0 mg/L (colorimetric) and 0.16 mg/L (fluorescence).

**Figure 10:**
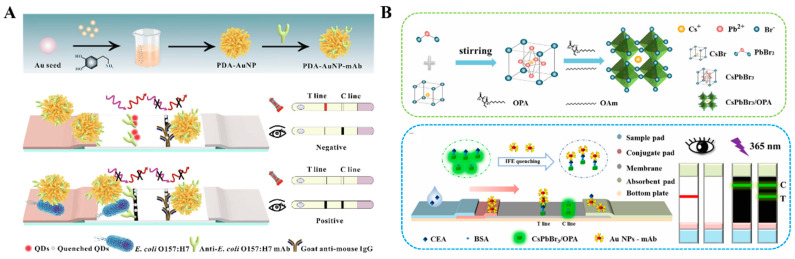
(**A**) Schematic illustration of a novel dual-mode LFIA based on PDA-AuNPs for the detection of *E. coli* O157:H7. (**A**) Preparation of PDA-AuNP-mAb probes. (**B**) Test principle of colorimetry/fluorescence dual-mode LFIA for the detection of *E. coli* O157:H7 [131]. Copyright 2023 Elsevier. (**B**) Schematic illustration of the synthesis process of CsPbBr_3_/OPA NCs. (**B**) CsPbBr_3_/OPA NCs and AuNP-based dual-readout LFIA for sensitive detection of CEA [134]. Copyright 2024 Elsevier.

In the aforementioned works, nanomaterials were used as the colorimetric and fluorescence signal tags. Moreover, AuNPs and their composites can serve as quenchers to reduce the fluorescence of luminescent materials modified on the nitrocellulose films [125,126,127,128,129,130]. For example, Shao et al. reported a dual-signal LFIA method for the detection of *E. coli* O157:H7 based on polydopamine (PDA)-modified AuNPs (PDA-AuNPs) (Figure 10A) [131]. PDA-AuNPs with a broad absorption band provided an obvious colorimetric signal. Meanwhile, PDA-AuNPs served as excellent quenchers to quench the fluorescence of QDs. Based on a similar principle, AuNP-modified MXene Ti_3_C_2_ was employed to construct a colorimetric/fluorescence LFIA platform for the detection of dexamethasone [132]. Zhang et al. presented a fluorescence quenching--based LFIA for dual-mode detection of CEA and carbohydrate antigens (CA153) using super-paramagnetic nanospheres as the quenchers to quench the fluorescence of antibody-conjugating Cy5 [133]. Recently, Wang et al. reported a dual-mode LFIA platform for CEA detection using amphiphilic polymer-capped CsPbBr_3_ perovskite nanocrystals as fluorescence reporters [134]. As illustrated in Figure 10B, CsPbBr_3_ nanocrystals capped with ctylamine-modified polyacrylic acids possessed high stability and bright fluorescence. AuNPs in the immunoprobes efficiently quench the green fluorescence of CsPbBr_3_ nanocrystals on the T line through the IFE effect. Besides these works, other colorimetric–fluorescence dual-signal LFIA methods with different signal tags have been proposed and their performances are shown in Table 2 [135,136,137,138,139].

**Table 2:** Performances of colorimetric–fluorescence dual-signal LFIA methods.

Signal Label	Target	Linear Range	Detection Limit	Ref.
PFTC6FQ@AuNP	CYFRA21-1	0–10 ng/mL	70 pg/mL	[114]
Au-Ag NCs@PEI-AuNPs	dicofol	8.45–94.79 and 1.36–19.92 ng/mL	4.16 and 0.62 ng/mL	[115]
SiO_2_@Au/QD	S1 protein	0.05–1000 ng/mL	1 and 0.033 ng/mL	[119]
MoS_2_@QDs	clothianidin	Not reported	120 and 2.58 pg/mL	[121]
AIENBs	CRP	8–100 and 0.2–100 mg/L	8 and 0.16 mg/L	[124]
AuNPs/C2-15-EmGFP	imidaclothiz	3.21–35.8 and 2.62–34.6 ng/mL	64 and 8 ng/mL	[126]
Au@PDA	carbendazim	7.21–945.55 and 3.56–246.67 ng/mL	3.21 and 1.36 ng/mL	[127]
APNPs	AC, CLE	0–4.0 ng/mL (AC), 0–10 ng/mL (CLE)	13 pg/mL (AC), 152 pg/mL (CLE)	[128]
ZnCdSe/ZnS@AuNPs	SBT	3.9–62.5 ng/mL	3.9 ng/mL	[129]
PD-AuNPs	*E. coli* O157:H7	97.7–12,500 CFU/mL	330 and 90.6 CFU/ mL	[131]
Ti_3_C_2_@Au	DXMS	0.05–0.8 μg/kg	1.8 and 1.3 ng/kg	[132]
CsPbBr_3_/OPA/AuNPs	CEA	0–1 ng/mL	027 and 23 pg/mL	[134]
APDA	Gen	0–1 ng/mL	1 and 0.5 ng/mL	[135]
PDQB	SARS-CoV-2	0.005–1 ng/mL	100 and 5 pg/mL	[136]
Si-Au/DQD	A29L	0.005–100 ng/mL	500 and 2.1 pg/mL	[137]
SAQDsRu	zearalenone	0.01–3 ng/mL	8 and 5.8 pg/mL	[138]
TRFNPs@HRP	ATC	0.2–100 ng/mL	80 pg/mL	[139]

Abbreviation: PFTC6FQ, a type of conjugated polymer; AuNP, gold nanoparticle; CYFRA21-1, cytokeratin 19 fragment; Au-Ag NCs, glutathione-capped Au-Ag bimetallic nanoclusters; PEI-Au NPs polyethyleneimine modified gold nanoparticles; SiO_2_@Au/QD, Au nanoparticles and quantum dots on SiO_2_; S1 protein, spike 1 protein of SARS-CoV-2; MoS_2_@QDs, molybdenum disulfide nanosheet-loaded quantum dots; AIENBs, aggregation-induced emission nanobeads encapsulated with red AIE luminogen; CRP, C-reactive protein; C2-15-EmGFP, fluorescent peptide tracer; PDA-AuNPs, polydopamine-modified AuNPs; AC, ractopamine; CLE, clenbuterol; APNPs, Prussian blue nanoparticle-coated gold nanoparticles; ZnCdSe/ZnS, ZnCdSe/ZnS quantum dots; SBT, sibutramine; Ti_3_C_2_@Au, MXene-Au nanoparticle; DXMS, dexamethasone in milk; CsPbBr_3_/OPA/AuNPs, Cs PbBr_3_ perovskite nanocrystals capped with amphiphilic polymer ligand of octylamine-modified polyacrylic acid and gold nanoparticles; CEA, carcinoembryonic antigen; APDA, polydopamine-coated AuNPs; Gen, gentamicin; PDQB, polydopamine@dual shell quantum dots nanobead; Si-Au/DQD, a multilayered SiO_2_-Au core dual-quantum dot shell nanocomposite; A29L, monkeypox virus antigen; SAQDsRu, *Staphylococcus aureus* biosynthesized quantum dots incorporating Ru(bpy)_3_^2+^; TRFNPs@HRP, polystyrene microsphere-encapsulated time-resolved fluorescent nanoparticles in conjugation with HRP; ATC, acetochlor.

### 2.2. Colorimetric–SERS Dual-Signal Immunoassays

**Figure 11:**
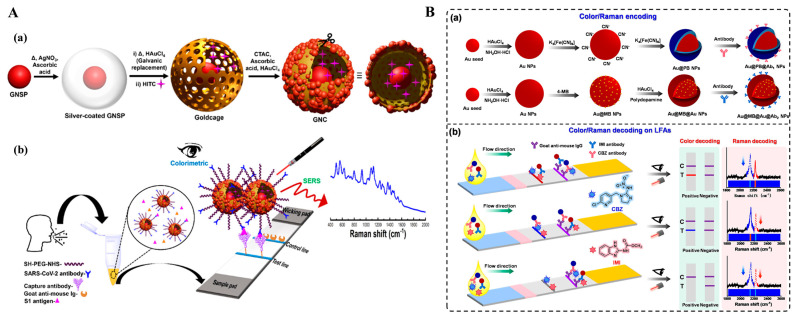
(**A**) Schematic illustration of (**a**) the synthesis of GNC and (**b**) the proposed colorimetric and SERS-based LFIA detection for S1 protein of SARS-CoV-2 [148]. Copyright 2024 American Chemical Society. (**B**) Schematic illustration of (**a**) the preparation process of the color and Raman-encoded nanoprobes. (**b**) The competitive LFA based on two types of nanotags for simultaneous detection of CBZ and IMI [151]. Copyright 2024 Elsevier.

As a powerful spectroscopic technique, SERS can enhance the inherent Raman scattering of analytes or signal reporters by 10^10^~10^11^-fold that were absorbed on the surface of nanostructured noble metals or hybridized nanomaterials [140]. SERS possesses the advantages of rapid detection speed, high sensitivity, and non-destructive characteristics, and has been widely used in trace analysis even at a single-molecule level [141]. However, SERS is still confronted with several drawbacks, including poor repeatability of substrate and low stability of signal data. To address these issues, SERS-based dual-mode biosensors have been developed to cross-verify by two modes and expand the corresponding detection ranges (Table 3) [142]. Among them, colorimetric and SERS dual-signal strategies can reduce the fluctuations in SERS detection and greatly increase the sensitivity of colorimetric analysis [143,144]. Au or Ag-based nanostructures can serve as not only the colorimetric reporters but also the SERS tags with high activity and controllable “hot-spot”, exhibiting the ability to develop colorimetric and SERS dual-mode LFIAs [145,146,147]. Recently, Atta et al. reported a colorimetric–SERS dual-mode LFIA platform for the determination of SARS-CoV-2 spike 1 (S1) protein with a plasmonic gold nanocrown (GNC) [148]. As presented in Figure 11A, GNC was engineered with a core–gap–shell nanostructure, and the gold shell was modified with external hotspot-rich Au nanospheres. Integrated with the LIFA platform, GNC produced a dark-blue colorimetric signal and an intense SERS signal that was generated by the Raman reporter embedded inside the interior nanogap. The colorimetric-based LFIA showed a detection limit of 91.24 pg/mL and that for the SERS-based method was 57.21 fg/mL. With a similar detection principle, Su et al. constructed a colorimetric–SERS dual-signal LFIA platform for the detection of clenbuterol using core–shell Au/Au nanostars as the multifunction tag [149]. Bai et al. applied Au@Ag-Au nanoparticles as the colorimetric and SERS dual-functional probes for the detection of cardiac troponin I [150]. It can be seen that the signal overlap in the Raman fingerprint region (400~1800 cm^−1^) and the similar color of SERS tags may limit the simultaneous detection of multiple targets within a single collection run. To resolve this problem, Wang et al. developed a colorimetric and SERS LFIA platform for the simultaneous detection of carbendazim (CBZ) and imidacloprid (IMI) using two types of nanoprobes with encoded color and Raman information [151]. As presented in Figure 11B, AuNPs coated by a PB shell (Au@PB NPs) possessed a characteristic Raman signal at 2151 cm^–1^ with a blue color. The other tag with a red color was prepared based on the immobilization of 4-mercaptobenzonitrile (4-MB) and the growth of a gold layer (Au@MB@Au NPs). Two types of nanotags were modified with different antibodies respectively, and then used in the competitive LFIAs. In the presence of CBZ and IMI, both color and Raman signals in a single were disappeared. This method for simultaneous quantitative analysis of CBZ and IMI showed a detection range of 3~9 ng/mL for the colorimetric assay and 0.1~12 ng/mL for the SERS assay.

**Figure 12:**
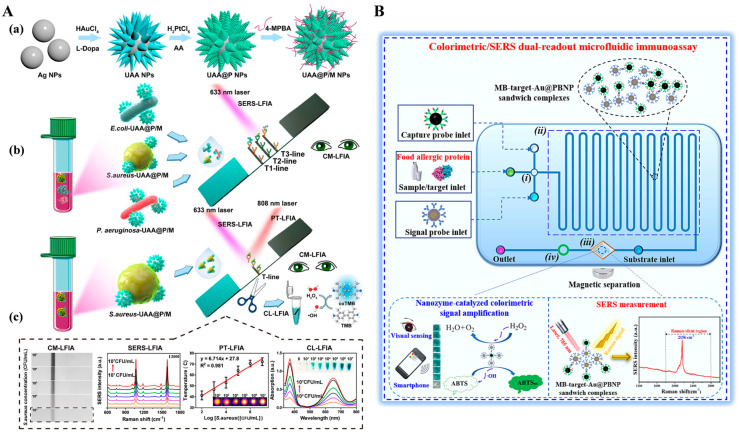
(**A**) Schematic illustration of (**a**) the synthesis process for multifunctional UAA@P/M; (**b**) the principle of the UAA@P/M-integrated LFIA for multiple bacterial discrimination; (**c**) the procedures of UAA@P/M-integrated LFIA for multimodal bacterial detection [153]. Copyright 2023 American Chemical Society. (**B**). Schematic illustration of the colorimetric/SERS dual-readout microfluidic immunoassay based on a bifunctional Au@PBNP nanozyme for detection of α-LA [155]. Copyright 2024 Elsevier.

In addition, SERS tags with enzyme-like catalytic ability can catalyze specific chromogenic reactions and lead to visible color changes, thus enabling colorimetric/SERS dual-signal detection. For example, He et al. reported a dual-mode LFA for *Campylobacter jejuni* detection by using platinum-coated gold nanorods (AuNR@Pt) with peroxidase mimicking and SERS enhancement properties as signal amplifiers [152]. Recently, Huang et al. developed a colorimetric, SERS, and photothermal LFIA method for the multiplexed detection of pathogenic bacteria using multifunctional urchin-shaped Au-Ag@Pt NPs (UAA@P NPs) (Figure 12A) [153]. In this study, UAA@P NPs were synthesized via a seed-mediated method and an in situ reduction procedure, and then modified with 4-mercaptophenylboronic acid (4-MPBA, UAA@P/M NPs). 4-MPBA selectively bound bacteria *S. aureus* by reaction with the peptidoglycan, lipopolysaccharide, and glycoprotein on the surface of bacteria to form the boronate bond. When the solution was loaded onto the sample pad of the strip, UAA@P/M NPs-labeled *S. aureus* was captured on the T-line. UAA@P/M NPs provided a strong SERS signal and converted the light energy into thermal that could be recorded by a thermal meter. Moreover, UAA@P/M NPs catalyzed the oxidation of TMB to realize colorimetric detection. However, antibodies or other biomacromolecules in practical samples may generate complex and unrecognizable Raman peaks at the fingerprint region (<1800 cm^−1^), leading to low selectivity and high background signal [154]. To overcome this shortcoming, Zhang et al. synthesized core–shell gold@Prussian blue nanoparticle (Au@PBNP) with high peroxidase-like activity and a unique Raman stretch vibrational peak for dual-mode detection of food allergic protein [155]. As shown in Figure 12B, alpha-lactalbumin (α-LA) in food samples was captured and then labeled in the microfluidic immunoassay. The Au@PBNP nanozyme catalyzed the oxidation of ABTS into green-colored ABTSox in the presence of H_2_O_2_. PB on the surface of Au@PB contained cyanide (CN^−^), which generated an enhanced stretch vibrational peak (2156 cm^−1^) at the Raman-silent region (1800–2800 cm^−1^).

### 2.3. Fluorescence–SERS Dual-Signal Immunoassays

**Figure 13:**
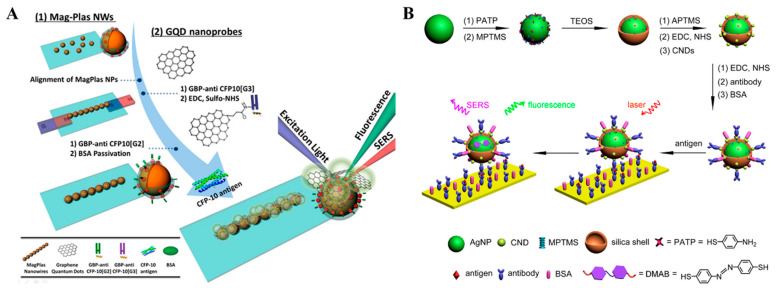
(**A**) Schematic illustration of a dual-mode immunoassay for CFP-10 detection using GQD labels and MagPlas nanoparticles [160]. Copyright 2016 American Chemical Society. (**B**) Schematic illustration of the construction of bifunctional CND-modified Ag/PATP@SiO_2_ tag for fluorescence and SERS immunoassays [165]. Copyright 2016 American Chemical Society.

The combination of fluorescence and SERS can enable biosensors with the merits of two techniques and overcome their respective drawbacks including the photobleaching of dyes and the long collection time of SERS assay [156]. Fluorescence and SERS dual-mode biosensors have been popularly used to determine various targets, such as metal ions, proteins, and so on (Table 3) [157]. Au and Ag nanomaterials with LSPR properties, such as nanospheres, nanorods, and alloy nanoshells, can be used as the SERS substrates to amplify the characteristic signals of Raman reporters modified on the substrate surface. Moreover, it has been demonstrated that the fluorescence emissions of dyes can be enhanced when they are immobilized on the surface at a specific distance. This fluorescence-enhanced phenomenon is known as metal-enhanced fluorescence (MEF) or surface-enhanced fluorescence [158]. For this view, Kim et al. fabricated a dual-functional tag with both SERS and MEF properties for fluorescence and SERS dual-signal immunoassays, in which polyelectrolytes were used to separate Ag-coated silica beads and fluorophores [159]. Graphene quantum dots (GQDs) can provide stable fluorescence and SRES signals because of their excellent photoluminescence and electronic properties. Zou et al. reported a dual-mode immunoassay platform with GQD labels and linearly aligned magneto-plasmonic (MagPlas) nanoparticles (NPs) [160]. As presented in Figure 13A, the superparamagnetic Fe_3_O_4_ NPs were coated with gold nanostructures and further assembled into 1D MagPlas nanochains. When the antibody-modified GODs were immobilized on the surface of MagPlas nanochains, GQD showed a D band and a G band at 1358 and 1579 cm^–1^ for Raman spectroscopy, respectively. Meanwhile, the fluorescence of GODs was enhanced via the MEF effect. 

Although Au and Ag nanomaterials have been popularly used as quenchers, dual-functional labels with fluorescence and SRES signals can be fabricated by appropriately adjusting the distance between nanomaterials and fluorophores [161]. For example, silica nanospheres were used to encapsulate fluorophores (or SRES substrates) and then modified with SRES substrates (or fluorophores) [162,163,164]. Zhang et al. developed a fluorescence and SRES immunoassay platform by the assembly of carbon nanodots (CNDs) and Ag@SiO_2_ as the bifunctional tags [165]. The synthesis procedure of CND-decorated Ag@SiO_2_ is presented in Figure 13B. The thickness of the silica shell was optimized to retain the strong fluorescence of CNDs and the strong signal of SRES tags. Raman reporter *p*-aminothiophenol (PATP) was converted into 4,4′-dimercaptoazobenzene (DMAB) through a photocoupling reaction under the illumination of a laser. The in-situ-formed DMAB produced high characteristic Raman shifts different from PATP. The detection limits of the fluorescence and SRES dual-mode immunoassays for goat-anti-mouse IgG detection were 100 ng/mL and 2.5 ng/mL, respectively.

**Table 3:** Performances of colorimetric–SERS and fluorescence–SERS dual-signal immunoassays.

Detection Mode	Signal Label	Target	Linear Range	Detection Limit	Ref.
Color–SERS	AuNS@Ag	*S. aureus*	1–10^7^ and 1–10^5^ CFU/mL	1 CFU/mL	[145]
	HITC@GNC	S1 protein	100–1000 and 0.0001–10 ng/mL	91.24 and 0.05721 pg/mL	[148]
	Au/Au nanostar	clenbuterol	0–1 ng/mL	5 and 0.05 ng/mL	[149]
	Au@Ag NPs	cTnI	5–50 and 0.9–50 ng/mL	4.5 and 0.09 ng/mL	[150]
	Au@PB NPs	CBZ	2–10 and 0.05–0.5 ng/mL	1.27 and 0.04 ng/mL	[151]
	AuNR@Pt	*C. jejuni*	10^2^–10^6^ and 10^2^–5 × 10^6^ CFU/mL	75 and 50 CFU/mL	[152]
	UAA@P NPs	*S. aureus*	100–10^7^ and 10–10^7^ CFU/mL	18 and 3 CFU/mL	[153]
	Au@PBNP	α-LA	1–600 and 0.2–600 ng/mL	283 and 11 pg/mL	[155]
	OMQ NPs	IgG	1–10^12^ fM	1 fM	[158]
FL–SERS	GQD	TB	0.001–1000 ng/mL	51.1 fg/mL	[160]
	CNDs/Ag@SiO_2_	IgG	0.01–100 μg/mL	100 and 2.5 ng/mL	[165]

Abbreviation: AuNS@Ag, silver-coated gold nanostar; *S. aureus*, *Staphylococcus aureus*; HITC, 1,3,3,1′,3′,3′-hexamethyl-2,2′-indotricarbocyanine iodide; cTnI, cardiac troponin I; Au@PB NPs, Prussian blue coated on AuNPs; CBZ, carbendazim; AuNR@Pt, platinum coated gold nanorods; *C. jejuni*, *Campylobacter jejuni*; UAA@P NPs, urchin-shaped Au-Ag@Pt nanoparticles; Au@PBNP, gold@Prussian blue nanoparticles; α-LA, alpha-lactalbumin; OMQ NPs, organic–metal–quantum dot hybrid nanoparticles; FL, fluorescence; GQD, graphene quantum dot; TB, tuberculosis antigen; CNDs, carbon nanodots.

## 3. Electrochemical Dual-Signal Immunoassays

Electrochemical biosensors have the unique characteristics of small size, high sensitivity, and easy portability [166]. According to the type of output signal, conventional electrochemical techniques include voltammetry, amperometry, potentiometry, and impedance spectroscopy. Recently, ECL and PEC methods based on the combination of electric and optical conversion processes have been developed for immunoassays. In this Section, electrochemical–ECL and electrochemical–PEC dual-signal immunoassays are discussed.

### 3.1. Electrochemical–PEC Dual-Signal Immunoassays

The PEC process involves the photo-to-electric conversion at an electrode/electrolyte interface under the illustration of applied light. Benefiting from two completely separated forms of excitation source and detection signal, PEC-based bioanalysis shows distinct advantages of high sensitivity, low background, simple operation, and ease of miniaturization. Therefore, PEC biosensors have been considered as the next generation of electrochemical methods for the ultrasensitive detection of disease markers. However, most of PEC immunoassays are conducted in a single–signal detection format and consequently suffer from weak external anti-interference ability and relatively high background and false-negative or -positive results, especially for the assays of complex samples. Therefore, PEC detection was combined with electrochemical analysis to improve the sensitivity and reliability and simplify the operation procedure [167].

**Figure 14:**
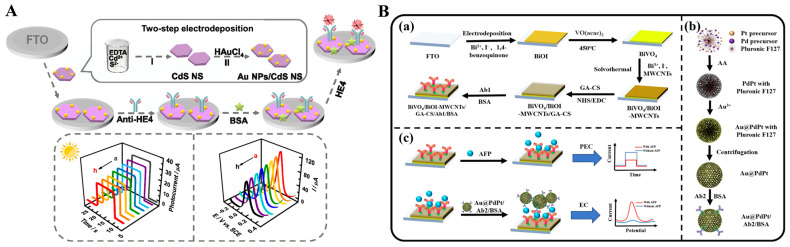
(**A**) Schematic illustration of AuNPs/CdS NS-based label-free dual-mode immunoassay for HE4 detection [168]. Copyright 2022 American Chemical Society. (**B**) (**a**) Fabrication process of the immunosensor, (**b**) synthesis process of Au@PdPt nanospheres, (**c**) AFP detection process of the immunosensor [170]. Copyright 2024 Elsevier.

The formation of immunocomplexes on the electrode surface can produce steric hindrance and block the diffusion of redox species in solution to the electrode surface, leading to a decrease in the electrochemical and PEC signals. Wei et al. reported a dual-mode electrochemical–PEC immunosensor for label-free detection of human epididymis protein 4 (HE4) by using SPR-promoted AuNPs/CdS nanosheet (CdS NS) heterostructures (Figure 14A) [168]. In this work, AuNPs improved the conductivity of CdS NS and promote the separation and transfer of charges under light irradiation, eventually enhancing the electrochemical and PEC signals. When HE4 was captured by antibodies, the diffusion of AA or [Fe(CN)_6_]^3−^/[Fe(CN)_6_]^4−^ was blocked, resulting in the decline of electrochemical and PEC signals. The developed dual-mode immunoassays for HE4 detection achieved a wide linear range of 0.01–200 ng mL^−1^ electrochemical assay and 0.01–100 for the PEC assay.

Nanocomposites with excellent electrochemical activity can be used as signal labels for immunoassays [169]. Wu et al. developed a label-free electrochemical and PEC dual-mode immunoassay for alpha-fetoprotein (AFP) detection based on BiVO_4_/BiOI-MWCNTs and Au@PdPt [170]. As illustrated in Figure 14B, the conductive glass FTO was modified with BiVO_4_/BiOIMWCNTs photoactive materials and antibodies sequentially. Due to the insulating features of proteins, antigens captured by antibodies impeded the electron communication between the photoelectrode and electron donor AA due to the steric hindrance effect, realizing the label-free PEC detection. Then, detection antibody-modified Au@PdPt was added to label AFP, producing a greatly amplified voltammetric signal for electrochemical detection.

### 3.2. Electrochemical–ECL Dual-Signal Immunoassays

**Figure 15:**
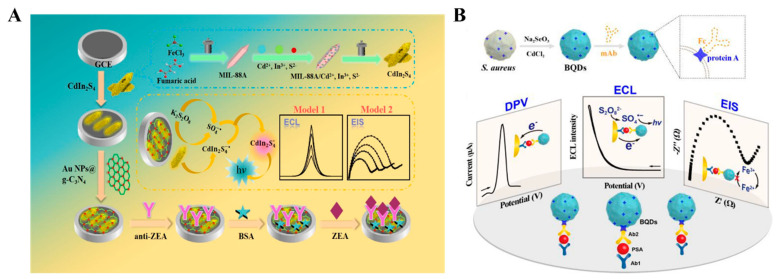
(**A**) Schematic illustration of an EC and PEC immunoassay for zearalenone analysis based on immunocomplex-induced steric hindrance effect [174]. Copyright 2024 Elesvier. (**B**) Schematic illustration of an EC and PEC immunoassay for PSA detection based on BQDs [175]. Copyright 2020 American Chemical Society.

ECL refers to a process of converting electrical energy into luminescence through redox reactions occurring on the electrode surface. ECL immunoassays have gained tremendous interest by virtue of cost-efficient equipment, low background signal, and ease of use. The integration of electrochemical and ECL detection techniques not only incorporates the electrochemical property but also avoids the use of an external light source [171,172,173]. As an example, Hao et al. reported an electrochemical–PEC dual-signal immunosensor for zearalenone analysis based on an immunocomplex-induced steric hindrance effect (Figure 15A) [174]. In this study, CdIn_2_S_4_ was used as the NIR ECL emitter and AuNPs@g-C_3_N_4_ was employed to accelerate the electron transmission and amplify the ECL signal. After the immunoreaction on the electrode surface, the formed antigen–antibody complexes blocked the diffusion of redox species onto the electrode by steric hindrance, leading to a decrease in the ECL signal and an increase in electrochemical impedance response.

QDs have been widely used in immunoassays due to their excellent electrochemical and ECL properties. However, the conjugation of nanomaterials including QDs with antibodies may undergo complicated and laborious modification processes. Wang et al. reported an electrochemical–ECL dual-signal immunoassay platform for the detection of *S. aureus* with the aid of biosynthesized QDs (BQDs) without time-consuming and labor-intensive functionalization processes (Figure 15B) [175]. Protein A expressed on the surface of *S. aureus* specifically bound to the Fc region of the antibody. BQDs produce a high DPV peak of around −0.8 V due to the electrochemical oxidation of metal ions in BQDs. The formation of sandwich immunocomplexes on the electrode surface impeded the electron transfer kinetics of [Fe(CN)_6_^3−^/[Fe(CN)_6_^4−^ due to the insulating and steric hindrance effects. Electrochemical impedance spectroscopy (EIS) was used to record the increased interfacial electron transfer resistance (R_et_). Furthermore, BQDs generated a strong ECL signal with K_2_S_2_O_8_ as the co-reactant. The method achieved the detection limits of 0.98 pg mL^−1^ by differential pulse voltammetry (DPV), 3.82 pg mL^−1^ by EIS, and 6.86 pg mL^−1^ by ECL for PSA detection.

## 4. Optical and Electrochemical Dual-Signal Immunoassays

In recent years, the translation of immunoreactions into detectable optical and electrical signals has become one of the most popular study topics [176,177]. Under the intelligent design and elaborate selection of nanomaterials, optical and electrochemical methods can be complementary with minimized errors from detection conditions and operations.

### 4.1. Colorimetric–Electrochemical Dual-Signal Immunoassays

**Figure 16:**
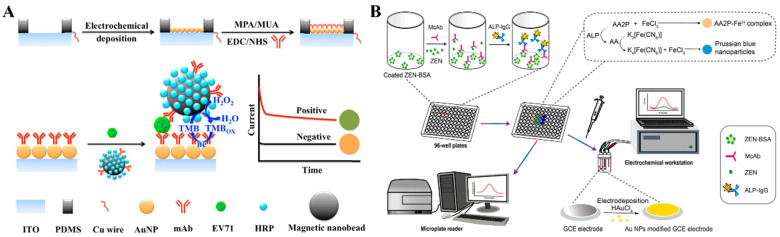
(**A**) Schematic illustration of a pretreatment-free colorimetric and EC immunoassay for the detection of EV71 using dual-functional magnetic nanobeads [187]. Copyright 2018 Elsevier. (**B**) Schematic illustration of a colorimetric and EC immunoassay for the detection of ZEN based on the enzymatic product-triggered formation of PB NPs [188]. Copyright 2022 Elsevier.

Colorimetric immunoassays show potential in point-of-care detection due to the naked-eye readout detection of antigens. However, they may suffer from low sensitivity and narrow linear range. The combination of electrochemical detection with colorimetric assay has achieved sensitive, automatic, and rapid on-site analysis (Table 4) [178,179,180]. Enzymes can catalyze the conversion of some substrates into the corresponding products with colorimetric and electrochemical responses [181,182]. Nanomaterials can be used as nanocarriers to load multiple antibodies and enzymes to further amplify the signals [183]. Magnetic nanobeads have attracted considerable attention in immunoassays due to the merits of facile manipulation and separation [184,185,186]. Hou et al. developed a pretreatment-free colorimetric and electrochemical immunoassay platform for the detection of enterovirus 71 (EV71) with the aid of magnetic nanobeads (Figure 16A) [187]. In this study, magnetic nanobeads were modified with EV71 mAb and HRP simultaneously. After the sequential immobilization of EV71 and dual-functional magnetic nanobeads, sandwich-type immunocomplexes were formed on the surface of a AuNP-modified ITO electrode. HRP on the magnetic nanobeads catalyzed the oxidation of TMB into colored TMBox, providing a colorimetric signal. Meanwhile, TMBox was electrochemically reduced on the electrode surface, enabling the chronoamperometric detection of EV71. The proposed dual-signal method achieved the detection limits of 1.0 ng/mL (colorimetric) and 0.01 ng/mL (electrochemical). In addition, Shang et al. reported a dual-signal immunoassay platform for colorimetric and electrochemical detection of zearalenone (ZEN) based on the enzymatic product-triggered generation of Prussian blue nanoparticles (PB NPs) (Figure 16B) [188]. In this work, ALP catalyzed the hydrolysis of AAP into AA, and then K_3_[Fe(CN)_6_] was reduced by AA into K_4_[Fe(CN)_6_], which interacts with Fe^3+^ to produce PB NPs. The produced PB NPs showed a blue color and AAP reacted with Fe^3+^ to form an orange-yellow AAP–Fe^3+^ complex. When ZEN was determined in the competitive immunoassay, the solution color changed from blue to orange-yellow and the absorbance spectra of PB NPs at 700 nm were recorded by a UV–vis spectrometer. Meanwhile, the transformation of K_3_[Fe(CN)_6_] into PB NPs led to a decrease in K_3_[Fe(CN)_6_] content in the solution, which could be determined by DPV for the electrochemical detection of ZEN.

**Figure 17:**
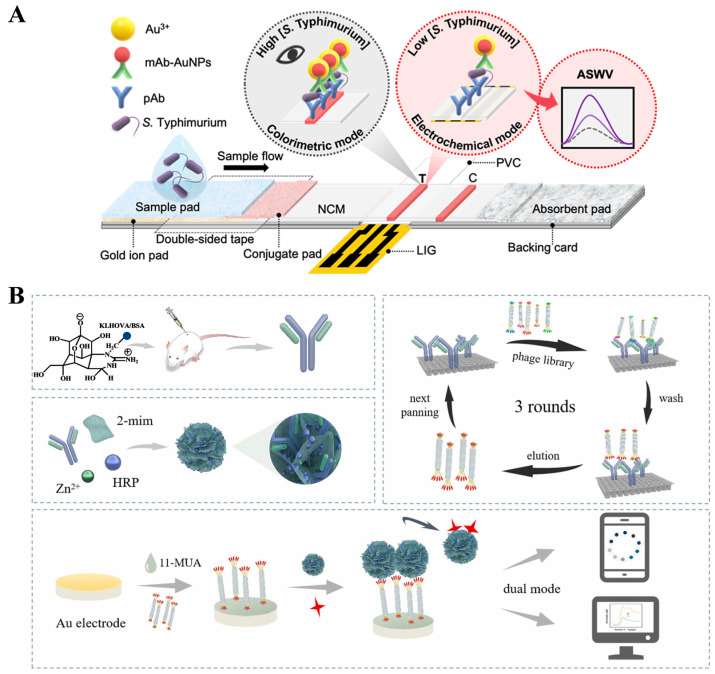
(**A**) Schematic illustration of the LIG–LFIA strip for colorimetric/electrochemical detection of *S. typhimurium* [189]. Copyright 2023 American Chemical Society. (**B**) Schematic illustration of a dual-mode immunoassay for EC and colorimetric detection of TTX based on biomineralized HRP/anti-TTX mAb@ZIF-8 [190]. Copyright 2023 Elsevier.

Nanomaterials with optical and electrochemical activity have aroused widespread interest in the development of dual-signal immunosensors [179,185]. Most LFIA strips can only provide qualitative or semiquantitative analysis based on the colorimetric signal, and the introduction of other readout signals can improve LFIA performance. Preechakasedkit et al. reported a colorimetric and electrochemical LFIA platform for the detection of *Salmonella typhimurium* (*S. typhimurium*) with single-step sample loading (Figure 17A) [189]. In this study, laser-induced graphene was used as the working electrode in the LFIA strip. Monoclonal antibody (mAb)-modified AuNPs were used to label *S. typhimurium*. An AuNP-catalyzed Au^3+^ electrodeposition strategy was employed to enhance the colorimetric response. Then, the anodic square wave voltammetry (ASWV) was utilized to measure the stripping signal of Au^0^ for electrochemical detection of *S. typhimurium*. To avoid the complicated modification of recognition elements and signal probes, biomineralization methods were applied for the co-encapsulation of antibodies and enzymes into nanomaterials. Li et al. reported a dual-mode immunoassay platform for colorimetric and electrochemical detection of tetrodotoxin (TTX) (Figure 17B) [190]. In this study, ZIF-8 was employed to in situ capsulate anti-TTX mAb and HRP (HRP/anti-TTX mAb@ZIF-8) as the recognition element and signal probe. A phage-displayed mimic peptide was screened to specifically bind with the anti-TTX mAb. TTX in samples competed with the mimic peptide to react with HRP/anti-TTX mAb@ZIF-8. The increase in TTX concentration led to a decrease in the amount of HRP/anti-TTX mAb@ZIF-8 to catalyze the oxidation of TMB into blue-colored TMBox. In the electrochemical method, the poorly conductive HRP/anti-TTX mAb@ZIF-8 captured by peptide-modified electrode reduced the oxidation signal of TMB due to the steric hindrance effect. 

### 4.2. Fluorescence–Electrochemical Dual-Signal Immunoassays

**Figure 18:**
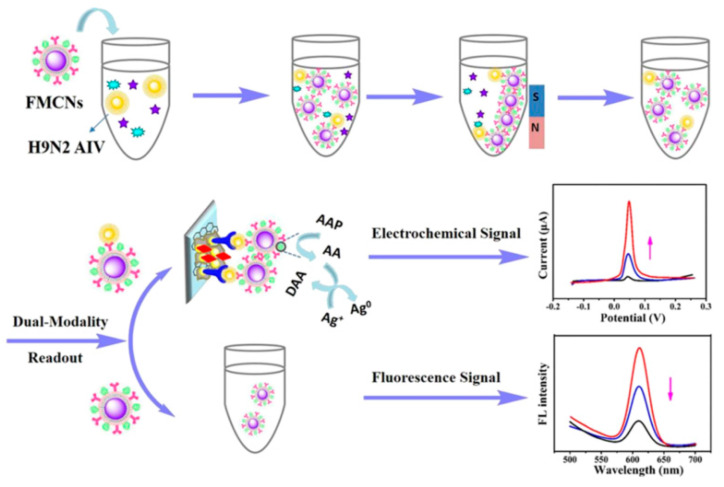
Schematic illustration of the fluorescent-magnetic-catalytic nanosphere-based EC and fluorescence immunoassay for the detection of H9N2 AIV [195]. Copyright 2019 American Chemical Society.

Fluorescence–electrochemical dual-signal immunoassays have been reported for the detection of targets with excellent selectivity and sensitivity [191,192]. Due to the stripping behavior of metal components and their excellent photoluminescence properties, quantum dots have been widely used as signal labels for fluorescence and electrochemical bioassays, including CdS, ZnS, and PbS [193]. Chopra et al. reported a fluorescence and electrochemical immunoassay platform for the detection of diabetic marker glycated hemoglobin (HbA1c) using CdTe QDs as the dual-functional tracers [194]. Anti-HbA1c antibody-modified CdTe QDs were used to label HbA1c, which was immobilized on the capture antibody-modified microtiter plate. QDs, directly providing a fluorescence signal, were dissolved in an acid solution, and the concentration of Cd^2+^ was determined by anodic stripping voltammetry. Peng et al. reported the fluorescence–electrochemical dual-signal immunoassays with CdSe/ZnS QD and ALP-modified *γ*-Fe_2_O_3_ nanoparticles as the fluorescent-magnetic-catalytic nanospheres (FMCNs) [195]. As shown in Figure 18, mAb-modified FMCNs specifically captured the H9N2 avian influenza virus (H9N2 AIV) from complex samples. Then, the antigen/FMCNs complexes were separated and enriched with the assistance of an external magnetic field and further captured by the polyclonal antibody (pAb)-modified ITO electrode. Then, ALP in the FMCNs catalyzed silver deposition reaction and metallic silver was quantitatively determined by linear sweep voltammetry.

### 4.3. Colorimetric–PEC Dual-Signal Immunoassays

**Figure 19:**
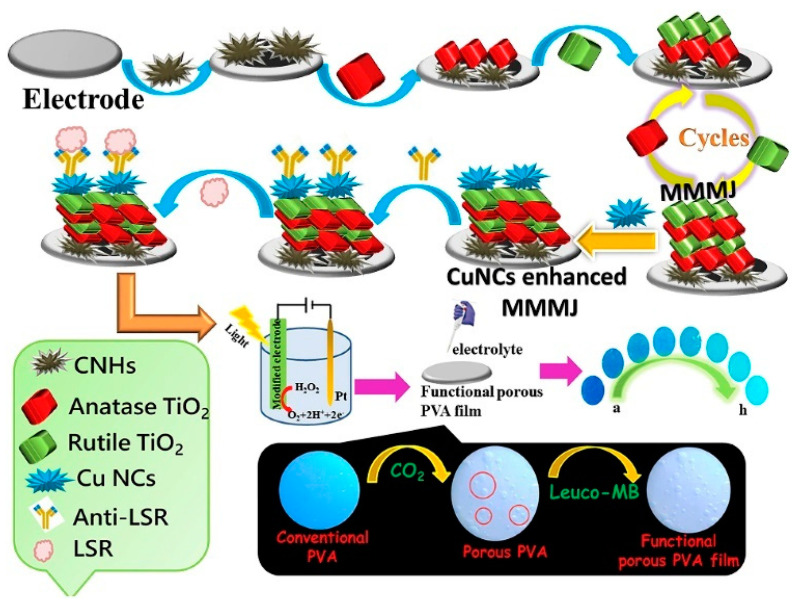
Schematic illustration of a PEC–colorimetric immunoassay for the detection of LSR based on a multiple mixed TiO_2_ mesocrystal junction [197]. Copyright 2020 Elsevier.

The combination of PEC detection with colorimetric analysis can improve the accuracy and reliability of bioassays (Table 4) [196]. In label-free immunoassays, the formation of immunocomplexes on the PEC electrode reduced the photocurrent by steric hindrance. Chen et al. reported a colorimetric/PEC immunoassay for the detection of lipolysis-stimulated lipoprotein receptor (LSR) based on a multiple mixed TiO_2_ mesocrystal junction (MMMJ) [197]. As shown in Figure 19, MMMJ was assembled on the ITO electrode through the repeated deposition of different phases of TiO_2_ mesocrystals (Anatase and Rutile) and was further decorated with Cu nanoclusters (CuNCs). The resulting electrode catalyzed the decomposition of H_2_O_2_ and produced a high photocurrent signal. Then, the solution was dropped on the Leuco-MB-modified film, and H_2_O_2_ in the solution oxidized the Leuco-MB, resulting in the color change. Once the antigen was captured by the antibody-functionalized electrode, the decomposition of H_2_O_2_ was suppressed. This will lead to the decline of the colorimetric and photocurrent signals.

**Figure 20:**
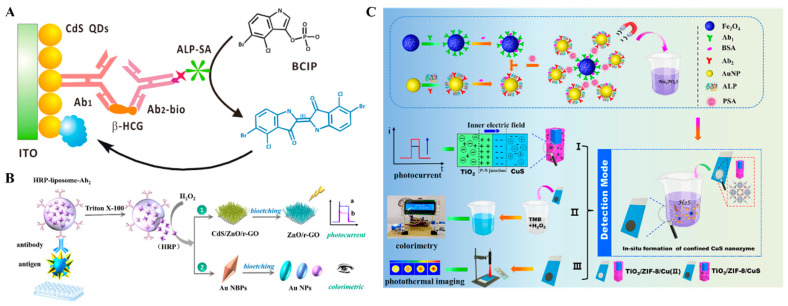
(**A**) Schematic illustration of a PEC and colorimetric immunoassay-based enzymatic biocatalytic precipitation [201]. Copyright 2022 American Chemical Society. (**B**) Schematic illustration of a bioetching-triggered PEC and colorimetric immunoassay using HRP-encapsulated nanoliposomes [202]. Copyright 2022 American Chemical Society. (**C**) Schematic illustration of the proposed multi-mode PEC immunoassays based on TiO_2_/ZIF-8/Cu(II) [203]. Copyright 2024 Am.

Enzymatic reactions can be employed to influence the photo-responsive system and regulate (enhance or decrease) the photocurrent signals [198,199]. For example, enzymes or nanozymes can catalyze the conversion of substrates (e.g., including 4-chloro-1-naphthol and hydroquinone) into non-conductive precipitates that can be in situ deposited on the photoelectrode surface, blocking the electron transfer and amplifying the PEC signal [200]. Zhang et al. reported the colorimetric and PEC dual-mode immunoassays based on enzymatic biocatalytic precipitation (Figure 20A) [201]. In this study, ALP was utilized as the enzymatic label to catalyze the hydrolysis of 5-bromo-4-chloro-3-indoyl phosphate (BCIP) into insoluble and blue-colored indigo products. Meanwhile, the precipitation formed on the surface of the CdS QDs/ITO electrode blocked the interfacial mass and electron transfer, thereby resulting in a decrease in the photocurrent signal. Wei et al. developed a bioetching-triggered colorimetric and PEC immunoassay platform for multiplex detection of ochratoxins using HRP-encapsulated nanoliposomes (Figure 20B) [202]. In this work, the PEC system was established based on the CdS/ZnO nanorod arrays/reduced graphene oxide. After the formation of the immunocomplexes on the wells, the encapsulated HRP molecules were released from nanoliposomes in the presence of Triton X-100. In the presence of H_2_O_2_, HRP catalyzed the bioetching of Au nanobipyramids (Au NBPs) and CdS, leading to multiple color changes and a decrease in photocurrent. However, this method was performed in a “signal-off” mode. Furthermore, Meng et al. developed multi-mode colorimetric and PEC immunoassays for PSA detection based on the enzymatic catalysis-induced MOF-confined plasmonic nanozyme (Figure 20C) [203]. In this work, ALP-labeled immunomagnetic beads were used to construct a split-type immunoassay platform for PSA detection. ALP catalyzed the hydrolysis of sodium thiophosphate solution to release H_2_S, which reacted with Cu^2+^ to generate CuS in ZIF-8. The formation of p-n heterojunctions (TiO_2_/ZIF-8/CuS) obviously increased their light-harvesting ability and improved the charge separation efficiency, leading to an enhanced PEC signal. In addition, CuS serving as a nanozyme catalyzed the chromogenic reaction of TMB in the presence of H_2_O_2_. The produced TiO_2_/ZIF-8/Cu(II) as a photothermal imaging probe showed high light-to-thermal conversion efficiency.

**Figure 21:**
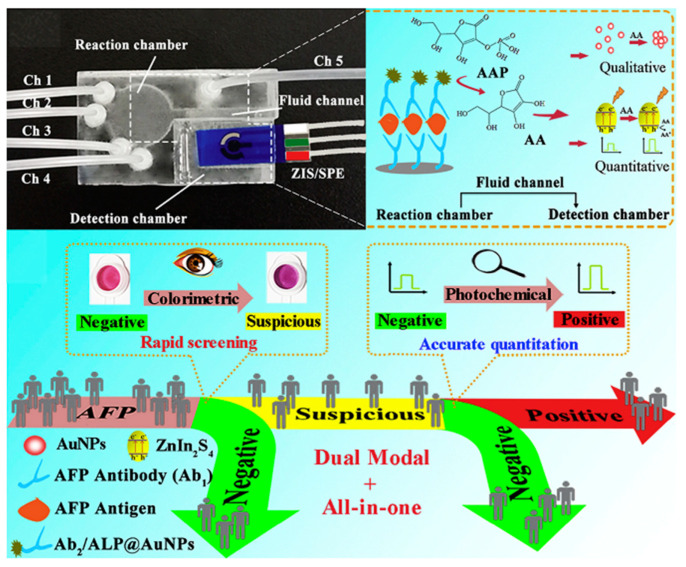
Schematic illustration of design and fabrication and the response mechanism of the 3D-printing “all-in-one” colorimetric and PEC dual-modal immunoassay [205]. Copyright 2020 Elsevier.

In PEC systems, electron donors/acceptors as the hole/electron-trapping species can greatly amplify the photocurrent signals and improve analytic performances. Therefore, it is possible to regulate the contents of electron donors/acceptors for developing dual-mode immunoassays. Enzymes-assisted strategies have been widely used to catalyze the hydrolysis of substrates to produce redox species and further trigger the change in optical or electrochemical signals [204]. Li et al. reported a colorimetric and PEC dual-mode all-in-one bioassay platform for AFP detection based on the ALP-enzymatic in situ generation of AA (Figure 21) [205]. All step-analysis working components were integrated together using automatic microfluidics. AA produced from ALP catalysis reduced cystine into cysteine, causing the aggregation of AuNPs with the color change from wine red to dark purple. In addition, the electron donor of AA scavenged the photogenerated holes and inhibits the recombinant of electron–hole pairs, obviously improving the PEC response.

### 4.4. Fluorescence–PEC Dual-Signal Immunoassays

**Figure 22:**
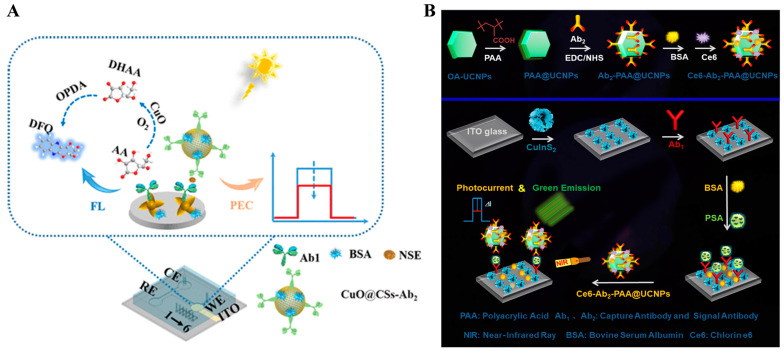
(**A**) Schematic illustration of the dual-mode microfluidic sensing platforms based on the CuO@CSs–Ab_2_ bioconjugate [207]. Copyright 2022 American Chemical Society. (**B**) Schematic illustration of the Ce_6_–Ab_2_–PAA@UCNP bioconjugates and the working process of the CuInS_2_ matrix-based dual-readout analyzing platform [208]. Copyright 2021 American Chemical Society.

Dyes in real samples may cause a high background signal during fluorescence analysis. The fluorescence/PEC dual-signal method is a feasible approach to overcome the above problems by enhancing accuracy and sensitivity [206]. Different from the traditional methods based on enzymatic production of electron donor AA, Wu et al. fabricated a dual-signal microfluidic sensing platform by using CuO nanozymes as multifunctional signal labels (Figure 22A) [207]. CuO NPs with an ascorbate oxidase-like property were loaded by carbon spheres (CuO@CSs). During the sandwich immunoreaction, antibody-modified CuO@CSs were immobilized on the PEC electrode to catalyze the oxidation of AA, leading to the quenching of the PEC signal. Then, the oxidation product DHA was reacted with OPD to form 3-(1,2-dihydroxyethyl) furo[3,4-b]quinoxaline-1-one (DFQ) with a strong fluorescence signal.

Near-infrared fluorescence visualization (NIR-FV)-based detection is a convenient way for bioanalysis, in which the signal can be observed by the naked eye. Han et al. reported NIR–FV and PEC-based immunoassays using chlorin e6 (Ce6)-modified UCNPs as the signal labels (Figure 22B) [208]. CuInS_2_ microflowers were used to modify the electrode and provide the original photocurrent in the presence of dissolved O_2_ as the electron acceptor. When antibodies and Ce6-modified UCNPs were captured by the electrode, the complexes as the poor conductors reduced the photocurrent intensity and Ce6 catalyzed the conversion of dissolved O_2_ into reactive oxygen species under 980 nm NIR irradiation. The decreased content of dissolved O_2_ in solution resulted in the decline of the PEC signal. In naked-eye NIR-FV mode, UCNPs serve as an illuminant body to generate visible green light. In other research, the fluorescence quantitative detection was integrated with the platform with 5-carboxyfluorescein-labeled helix peptide as the PSA substrate [209]. Besides the similar NIR-FV and PEC modes, PSA enzymatically cleaved the dye-labeled peptide, leading to the release of 5-carboxyfluorescein for the fluorescence assay.

**Table 4:** Performances of optical-electrochemical dual-signal immunoassays.

Detection Mode	Signal Label	Target	Linear Range	Detection Limit	Ref.
Color–EC	PBNPs	TRX1	10–50 ng/mL	9 and 6.5 ng/mL	[179]
	PBNPs	IgG	0.5–10 µg/mL	34 ng/mL	[180]
	ALP	CA125	5–1000 and 50–1000 U/mL	1.3 and 40 U/mL	[182]
	HRP-Ppy NPs	PSA	0.001–40 ng/mL	0.8 and 0.7 pg/mL	[183]
	PP1-MPs	MC-LR	Not reported	7.4 and 0.4 μg/L	[184]
	uPtNZs	GA	0.01–5 and 0.005–10 mg/mL	9.2 and 3.8 µg/mL	[185]
	H-AuNPs	BNP	0.001–0.2 and 5–25 ng/mL	0.03 and 80.3 pg/mL	[186]
	HRP-MPs	EV71	0.1–600 ng/mL	1 and 0.01 ng/mL	[187]
	ALP	ZEN	0.2–0.8 and 0.125–0.5 ng/mL	40 and 80 pg/mL	[188]
	Au-AuNP	*S. typhimurium*	0.1–10^8^ CFU/mL	1 CFU/10 mL	[189]
	HRP/ZIF-8	TTX1	10.5–380 and 0.1–1000 ng/mL	183 and 10 pg/mL	[190]
Color–PEC	CuNCs	LSR	0.001–10,000 pg/mL	1 fg/mL	[197]
	HRP@NL	OTA	0.001–5 ng/mL	1.7 and 0.7 pg/mL	[202]
	ALP-AuNP	PSA	0.001–10 ng/mL	0.41 and 0.16 pg/mL	[203]
	ALP	AFP	0.05–100 ng/mL	10 pg/mL	[205]

Abbreviation: EC, electrochemical; PEC, photoelectrochemical; PBNPs, Prussian blue nanoparticles; TRX1, thioredoxin 1; ALP, alkaline phosphatase; HRP, horseradish peroxidase; Ppy NPs, polypyrrole nanoparticles; PSA, prostate-specific antigen; PP1-MPs, phosphatase 1-conjugated magnetic particles; MC-LR, Microcystin; uPtNZs, urchin-like Pt nanozymes; GA, glycated albumin; H-AuNPs, Hemin-modified gold nanoparticles; BNP, brain natriuretic peptide; EV71, enterovirus 71; TTX, tetrodotoxin; Au-AuNP, gold-deposited gold nanoparticle; *S. typhimurium*, Salmonella enterica serovar typhimurium; ZEN, zearalenone; ZIF-8, zeolitic imidazolate framework-8; FMCNs, fluorescent-magnetic-catalytic nanospheres; H9N2 AIV, H9N2 avian influenza virus; CuNCs, Cu nanoclusters; LSR, lipolysis stimulated lipoprotein receptor; HRP@NL, HRP-encapsulated nanoliposomes; OTA, ochratoxin A; AuNP, gold nanoparticle; AFP, alpha-fetoprotein.

## 5. Conclusions

In the past few decades, with the significant advancements in biotechnology and nanotechnology, we have witnessed the rapid development of immunoassays in different fields. In order to improve detection accuracy, dual-signal immunoassays that can provide high-throughput and multi-level detection data have received increasing attention. This article provides a detailed and systematic summary of the progress made in dual-signal immunoassays. For optical dual-signal immunoassays, we systematically introduced the working principles of visualization and fluorescence immunoassays, and discussed the combination of SERS technology with other optical methods. Then, we summarized the integrated strategies of electrochemical, ECL, and PEC techniques in electrical dual-signal immunoassays. Finally, the advances in optical and electrochemical dual-signal immunoassays were summarized. Compared with the single–signal methods, dual-signal immunoassays have the advantages of low background signal, high accuracy and reliability, more available information, and wide detection range.

Despite the significant achievements, there is still relatively large room for the construction and application of immunoassays to achieve high sensitivity and selectivity. Firstly, nanozymes have been widely used to improve the detection performances of dual-signal immunoassays, but the specificity and catalytic activity of nanozymes are still poor in contrast to natural enzymes. Different strategies should be adopted to address these shortcomings, including optimizing the size, shape, composition, and surface modification of nanozymes. Secondly, most dual-signal immunoassays involve the use of functional nanomaterials as signal labels or electrode matrices, but the stability and homogeneity of nanomaterials should be enhanced. Therefore, there is a high demand to synthesize various nanomaterials with excellent electrical conductivity and high stability for practical applications. Thirdly, most of the reported dual-signal immunoassays are performed in laboratory studies, and the complexity of real-world applications may dramatically hinder their development. The combination of simple and low-cost signal readers, especially those based on microfluidic chips, LFIAs, or smart wearable devices, can expand the practical applications of dual-signal immunoassays.

## Data Availability

Data are contained within the article.
